# Complex Role of Circulating Triglycerides in Breast Cancer Onset and Survival: Insights From Two‐Sample Mendelian Randomization Study

**DOI:** 10.1002/cam4.70698

**Published:** 2025-02-17

**Authors:** Wu Zhang, Zhiru Li, Yuquan Huang, Jing Zhao, Shaowei Guo, Qian Wang, Sihan Guo, Qingxia Li

**Affiliations:** ^1^ Fourth Department of Oncology Hebei General Hospital Shijiazhuang China; ^2^ Graduate School North China University of Science and Technology Tangshan Hebei China; ^3^ Department of Pathology Shengjing Hospital of China Medical University Shenyang China; ^4^ Sixth Department of Oncology Hebei General Hospital Shijiazhuang Hebei China; ^5^ Department of Computer Science Durham University Durham UK; ^6^ Hebei Medical University Shijiazhuang Hebei China

**Keywords:** breast neoplasms, mendelian randomization analysis, triglycerides

## Abstract

**Background:**

Reducing the incidence of breast cancer and improving its prognosis have become significant challenges for the global public health sector. We aimed to investigate the role of circulating triglycerides in the occurrence and survival of patients with breast cancer, while focusing on the possible differential effects by molecular subtypes of breast cancer.

**Methods:**

We used a Mendelian randomization approach to analyze publicly accessible genome‐wide association study data, including triglyceride levels, breast cancer risk, and survival prognosis. We performed a two‐sample causality inference analysis using the inverse‐variance weighted method. We used both Mendelian randomization–Egger regression and weighted median methods for model verification. Heterogeneity was evaluated using Cochran's *Q* test, and sensitivity analyses were performed using the leave‐one‐out method, Mendelian randomization–Egger intercept test, and Mendelian Randomization Pleiotropy RESidual Sum and Outlier test.

**Results:**

The results revealed a negative causal relationship between triglyceride levels and overall breast cancer risk (odds ratio [OR] = 0.94, confidence interval [CI] = 0.89–0.99, *p* = 0.011), luminal A breast cancer risk (OR = 0.93, CI = 0.87–0.99, *p* = 0.014), and human epidermal growth factor receptor 2 (HER2)‐enriched breast cancer risk (OR = 0.84, CI = 0.73–0.96, *p* = 0.010). However, no statistically significant correlations were observed for the luminal B, luminal B HER2‐negative, and triple‐negative subtypes. Furthermore, triglyceride levels showed a positive causal relationship with the risk of survival prognosis in patients with estrogen receptor‐negative breast cancer (OR = 1.33, CI = 1.00–1.76, *p* = 0.047). However, no statistically significant impact was observed on the survival of patients with overall breast cancer or patients with estrogen receptor‐positive, HER2‐positive, and HER2‐negative breast cancer.

**Conclusions:**

The potentially complex role of circulating triglycerides in the incidence and survival of patients with breast cancer provides a new perspective on the heterogeneity of the effects of triglycerides on breast cancer, thereby promoting the development of precise medical strategies. Moreover, our findings contribute to an increased understanding of overall health among patients and clinicians alike.

**Abbreviations**
BCAC: Breast Cancer Association
CI: confidence interval
GWAS: Genome‐Wide Association Study
HER2: Human Epidermal Growth Factor Receptor 2
IV: instrumental variable
IVW: inverse‐variance weighted
MR: Mendelian randomization
OR: odds ratio
SNP: single‐nucleotide polymorphism
WM: weighted median

## 1 Background

Breast cancer imposes the greatest cancer burden on women worldwide and is the primary cause of cancer incidence and mortality among women. The age‐standardized incidence rate of breast cancer among women worldwide is approximately 48 per 100,000 population. Notably, data from 2020 revealed that breast cancer accounted for premature deaths of approximately 685,000 women worldwide [1, 2]. The most recent statistics from the American Cancer Society (ACS) show that breast cancer accounts for 32% of all newly diagnosed common tumors in women [3]. Consequently, reducing the incidence of breast cancer and improving its prognosis have become significant challenges for the global public health sector.

Breast cancer is not a singular disease entity but rather a complex collection of diseases distinguished by diverse pathological types and molecular characteristics [4]. Breast cancer is categorized into five molecular subtypes according to the presence or absence of estrogen or progesterone receptors and human epidermal growth factor receptor 2 (HER2) and Ki67 expression levels: luminal A, luminal B, luminal B plus HER2 overexpression, HER2‐enriched, and triple‐negative subtypes [5, 6, 7]. Breast cancers with different gene expression patterns exhibit unique biological features and clinical manifestations, which greatly enhance the heterogeneity of breast cancer [8]. This heterogeneity complicates the understanding of breast cancer pathogenesis and prognostic outcomes and presents new challenges for exploring breast cancer risk factors, prognostic risks, and their relationships with various metabolic factors.

In fact, not only does obesity increase the risk of breast cancer, but the adverse lifestyle factors that can contribute to obesity can also increase the risk of breast cancer and lead to a poor prognosis. Increasing evidence based on body mass index suggests that obesity is associated with increased breast cancer risk and decreased survival rates [9, 10]. Results of a meta‐analysis on the relationship between lifestyle and breast adenocarcinoma suggest that obesity increases the risk of developing breast cancer [11]. Chen et al. [12] showed that a high‐fat diet induces obesity‐associated intestinal flora to activate the mTORC1 signaling pathway, which stimulates the activation of polymorphonuclear myeloid‐derived suppressor cells and promotes the development of breast cancer.

However, body mass index does not comprehensively reflect an individual's overall or localized fat distribution, especially in terms of accurately depicting fat metabolism status. Triglycerides, also known as triacylglycerol, are the main lipid components and forms of stored energy in the body [13, 14]. Studies have shown that metabolic syndrome is independently associated with breast cancer, and triglycerides are not only one of the components of metabolic syndrome but also directly reflect the status of fat metabolism, participate in the metabolic reprogramming of tumor cells, and play a key role in the occurrence, development, and prognosis of breast cancer [15, 16, 17, 18, 19]. Scholars are interested in the abnormalities of triglycerides and the development and prognosis of breast cancer, so studies have been conducted on different aspects of triglycerides and breast cancer. Unfortunately, the results of these studies have not been consistent. Among others, Franky et al. [20] showed that blood triglyceride levels were significantly higher in breast cancer patients compared to patients with benign breast tumors, and that hypertriglyceridemia could be significantly controlled after treatment in breast cancer patients who achieved complete remission.

A study by Yadav et al. [21] focused on the differences in blood triglyceride levels in breast cancer patients before and after menopause and showed that regardless of menopausal status, blood triglyceride levels were higher in breast cancer patients than those in the control.

In contrast, there are fewer studies on the effects of hypertriglyceridemia and obesity on the occurrence and survival of different subtypes of breast cancer, and these results appear to differ from previous results. A prospective cohort study suggests that obesity is associated with poor prognosis only in patients with Luminal A breast cancer, but not in patients with breast cancer of other molecular subtypes [22]. In a retrospective study, hypertriglyceridemia was a protective factor for patients with HER2+ breast cancer [23]. Uncertainty about the causal relationship between circulating triglycerides and the risk of breast cancer and its five molecular subtypes, as well as the prognostic relationship for survival, may be due to the presence of various confounding factors.

Directly determining the causal relationship between the exposure of interest and the outcome is not possible using a traditional observational study design because of observed and unobserved confounding factors, as well as reverse causation and selection bias [24, 25]. Although randomized controlled trials are considered the gold standard for establishing causality, extensive human and material costs, significant time expenditures, and potential ethical issues limit their widespread application in clinical research [26]. In contrast, a research method known as “nature's randomized trial” or Mendelian randomization (MR) has gained increased attention [27]. This genetic approach to epidemiology is based on Mendel's Law of Independent Assortment, which considers genetic variations to be randomly distributed among individuals. Using extensive genome‐wide association study (GWAS) data, genetic variations have been used as instrumental variables (IVs) to simulate randomized controlled research methods and explore the causal relationship between the exposure of interest and outcome randomly [28, 29, 30]. This method solves the cost issue associated with randomized controlled trials and eliminates the influence of common confounders and selection bias characterized by observational studies.

Therefore, in this study, we used a two‐sample MR approach to investigate the causal relationship between circulating triglycerides and the risk and survival prognosis of patients with breast cancer and its subtypes. We aimed to provide a theoretical basis for exploring the profound mechanisms underlying the association between circulating triglycerides and the development of breast cancer, thereby promoting the advancement of precise medical strategies and scientifically accurate perceptions of health among the public.

## 2 Methods

### 2.1 Study Design

**FIGURE 1:**
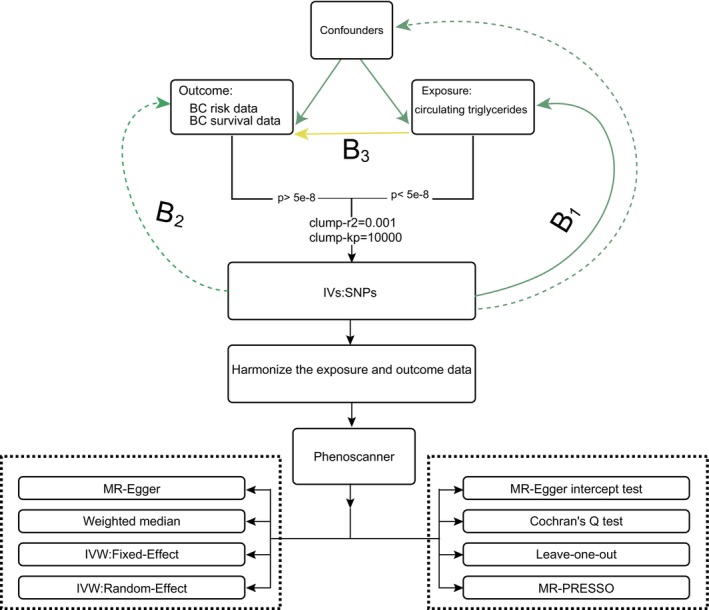
Triglycerides and breast cancer onset risk and survival. β1: The relationship between the instrumental variables and exposure. β2: The direct relationship between the instrumental variable and outcome. β3: The direct relationship between exposure and outcome. BC, breast cancer; IVs, instrumental variables; IVW, inverse‐variance weighted; MR, Mendelian randomization; MR‐PRESSO, Mendelian Randomization Pleiotropy RESidual Sum and Outlier; SNPs, single‐nucleotide polymorphisms. Solid lines indicate theorized paths; dashed lines indicate paths theorized to be non‐significant according to MR assumptions.

The central concept of MR involves the use of single‐nucleotide polymorphisms (SNPs) as IVs to evaluate the relationship between exposure factors and outcomes. This process must satisfy the following three key assumptions: (i) the strong relevance assumption, that is, a significant correlation exists between the IV and exposure factor of interest; (ii) the independence assumption, that is, the IV is unrelated to the confounding factors that simultaneously affect exposure and outcomes; and (iii) the exclusion restriction assumption, that is, the relationship between the instrumental variable and the outcome can only be produced by the exposure factor and not by other factors. Figure 1 illustrates the methods used in this study [31]. This study followed the Strengthening the Reporting of Observational Studies in Epidemiology using MR guidelines to ensure transparency and replicability [32]. This research has been conducted using published studies and consortia providing publicly available summary statistics. All original studies have been approved by the corresponding ethical review board, and the participants have provided informed consent. In addition, no individual‐level data was used in this study; therefore, no new ethical review board approval was required.

### 2.2 Circulating Triglyceride and Genetic Mutation Data

Information on circulating triglycerides was obtained from GWAS data based on the UK Biobank dataset provided by the Medical Research Council Integrative Epidemiology Unit (MRC‐IEU) consortium. This dataset includes 12,321,875 SNP sites and data from 441,016 participants (average age, 56.9 [range 39–73] years; women, 54.2%). According to the data, the median triglyceride level was 1.50 mmol/L, with an interquartile range of 1.11 mmol/L. This dataset was created by Richardson et al. [33] based on the European population in 2020 and mainly focuses on lipid levels. Detailed information regarding the dataset is available at https://gwas.mrcieu.ac.uk.

### 2.3 Breast Cancer Risk and Survival Data

The GWAS data regarding breast cancer risk mainly comprised two parts: overall breast cancer and breast cancer subtypes. All the breast cancer data from GWAS were obtained from 82 breast cancer association (BCAC) studies from more than 20 European countries. This dataset includes 133,384 patients with breast cancer and 113,789 controls. The types of cancers among patients with breast cancer include invasive cancer, in situ cancer, and cancers with unclear invasiveness. Breast cancer subtype data were obtained from 81 BCAC studies, including 106,278 patients with invasive breast cancer and 91,477 controls. The cancer subtypes identified in these patients included luminal A‐like, luminal B/HER2‐negative‐like, luminal B‐like, HER2‐enriched‐like, triple‐negative, and basal‐like (data on the subtypes are detailed in Data S1) [34].

Furthermore, we investigated the causal relationship between circulating triglycerides and breast cancer survival using GWAS data on breast cancer among Europeans that only considered HER2 and estrogen receptor (ER) status. This includes GWAS data on survival among patients with HER+ (4263 cases and 99,267 controls) and HER2‐ (7355 cases and 99,321 controls) breast cancer that was recently published in a Finnish database (http://www.fifinngen.fifi). We also included GWAS data on the overall breast cancer survival rates by ER status reported by Guo et al. [35] and published by BCAC studies in 2015. The ER breast cancer (survival) dataset mainly included data from survival studies on ER‐negative breast cancer (920 cases and 5961 controls). The ER+ breast cancer (survival) dataset focused on the survival rates of patients with ER‐positive breast cancer (1333 cases and 21,726 controls). The GWAS data on overall survival rates among patients with breast cancer included 37,954 European breast cancer samples, with 2900 deaths from breast cancer. All samples were subjected to detailed genetic typing, and approximately 12.94 million SNP sites were analyzed. All genetic data were based on the 19th edition of the human genome reference sequence (HG19/GRCh37) (Table 1).

**TABLE 1:** Cohort population data.

Variable	Cases (number)	Controls (number)	Resource	SNPs (number)	Population	Year
Triglycerides	441,016	—	ieu‐b‐111	12,321,875	European	2020
All BC Risk	133,384	113,789	BCAC	10,760,767	European	2020
BC Subtype	106,278	91,477	BCAC	9,965,310	European	2020
All BC Survival	2900	35,054	BCAC	12,940,150	European	2015
ER+ BC Survival	1333	21,726	BCAC	8,714,606	European	2015
ER‐ BC Survival	920	5961	BCAC	8,828,662	European	2015
HER2+ BC Survival	4263	99,267	FINN	16,379,180	European	2021
HER2‐ BC Survival	7355	99,321	FINN	16,379,314	European	2021

Abbreviations: BC, breast cancer; BCAC, breast cancer association Consortium; ER, estrogen receptor; FINN, Fire INventory from NCAR; HER2, human epidermal growth factor receptor 2.

### 2.4 Instrumental Variables

Prior to the MR analysis, we performed intricate data filtering and preprocessing. Our fundamental goal was to identify SNPs that are strongly correlated with circulating triglycerides. Accordingly, we favored SNPs presenting *p*‐values less than 5 × 10^−8^ to meet the robust instrument relevance prerequisite for MR. To fulfill the exclusion restriction supposition, we handpicked SNPs with *p*‐values exceeding 5 × 10^−8^ associated with the determined outcome. We designated *r* ^2^ as 0.001 and defined the span of the linkage disequilibrium region as 10,000 kb to eliminate the SNPs implicated in linkage disequilibrium. This strategy safeguards the genetic independence of the selected SNPs, thereby mitigating potential collinearity complications. Our chosen SNPs were uploaded to the Phenoscanner database, a compendium abundant with published human genotype–phenotype associations [36], to discard any SNPs that could potentially correlate with confounding elements, thereby meeting the independence requirement of MR.

### 2.5 Statistical Analysis

We harmonized shared SNPs between the exposure and outcome groups to eliminate biases potentially induced by a single SNP of varied genomic positioning, ensuring a valid representation of the exposure –outcome relationship. Next, we used the “TwoSampleMR” package in R software (version 4.2.1; R Foundation for Statistical Computing, Vienna, Austria) for two‐sample MR analysis, using inverse‐variance weighted (IVW) methodologies for our causal inference analysis [37]. In addition, we applied MR‐Egger regression and weighted median (WM) methods as ancillary tools for model evaluation. Considering pleiotropy, MR‐Egger regression relaxes the no‐horizontal‐pleiotropy condition, whereas the WM method allows up to 50% of the data from the MR analysis to stem from SNPs that are deemed invalid IVs, thereby promoting enhanced flexibility and robustness [38, 39]. Among these methods, the IVW technique primarily provided the results used to form our conclusions [40].

### 2.6 Heterogeneity and Sensitivity Analysis

To verify the robustness of our results, we assessed the heterogeneity of each instrumental variable by estimating the causal effects using Cochran's *Q* test. If the *Q* test results indicated disparate effects across the IVs, thereby revealing significant heterogeneity, we adopted a random‐effects model for the IVW method as our primary result. Conversely, if no heterogeneity is detected, we applied a fixed‐effects model for the IVW method. Potential directional pleiotropy was examined using the MR‐Egger intercept test. A significant nonzero MR‐Egger intercept value suggests the presence of directional pleiotropy. This indicates that our IVs, aside from influencing circulating triglycerides, may also affect other unobserved factors, thereby potentially biasing the estimate of the causal effect [41]. Subsequently, using the “MR‐PRESSO” R package, we identified and eliminated the outlying SNPs that could significantly influence the causal effect estimation based on horizontal pleiotropy (i.e., SNP outlier tests with *p*‐values < 0.05) [42]. After correcting for potential horizontal pleiotropy, we reevaluated the causal effects. Finally, we used the leave‐one‐out method for further sensitivity testing to evaluate the impact of individual SNPs on causal effects [41]. All statistical tests were two‐tailed. Given that our outcome was binary, we converted the effect estimates (β) into odds ratios (ORs) to provide a more intuitive depiction of the potential causal relationship and degree of association between circulating triglycerides and breast cancer risk and prognosis.

## 3 Results

### 3.1 Selection of Instrumental Variables

We used Phenoscanner to screen for SNPs with significant correlations with confounding factors, such as breast cancer, age at menarche, and hormone levels, and excluded some SNPs (rs13066793, rs7947951, rs11030107, rs3814883, rs9902027, and rs6517522) that were strongly associated with the exposure and outcomes. Ultimately, we selected 299 SNPs related to triglyceride levels from the initial set, all of which exhibited *p*‐values less than 5 × 10^−8^ (details in Data S2). While harmonizing circulating triglycerides with each breast cancer dataset, we omitted palindromic SNPs with intermediate allele frequencies to ensure the reliability of our results [43].

### 3.2 Circulating Triglycerides and Breast Cancer Risk

**FIGURE 2:**
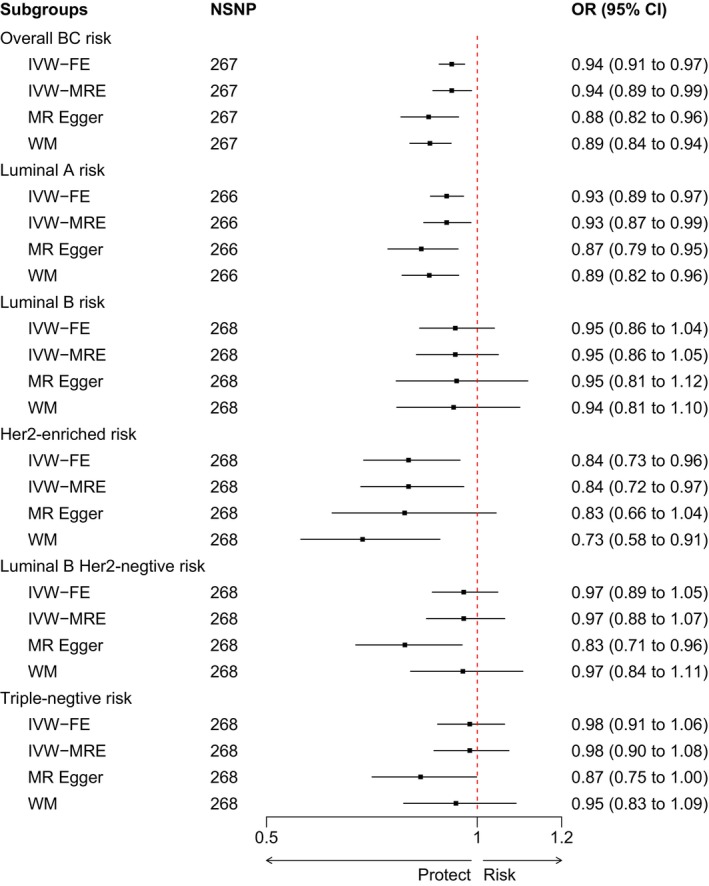
Forest plot of causal relationship between triglyceride levels and breast cancer risk using Mendelian randomization. BC, breast cancer; CI, confidence interval; FE, fixed‐effects model; HER2, human epidermal growth factor receptor 2; IVW, inverse‐variance weighted; MR, Mendelian Randomization; MRE, random‐effects model; NSNP, number of single‐nucleotide polymorphism; OR odds ratio; WM, weighted median.

The influence of circulating triglycerides on the risk of various types of breast cancer differed (Figure 2). Regarding overall breast cancer, the luminal A and HER2‐enriched subtypes showed a negative correlation between circulating triglycerides and breast cancer risk in both IVW models. According to the Cochran's *Q* test results (Data S3), heterogeneity was present in overall breast cancer and the luminal A subtype (*p* < 0.05), whereas the Cochran's *Q* test results for the HER2‐enriched type showed no significant heterogeneity (*p* > 0.05). As a result, the causal relationship between circulating triglycerides and overall breast cancer (OR = 0.94, CI = 0.89–0.99, *p* = 0.011) as well as the luminal A subtype (OR = 0.93, CI = 0.87–0.99, *p* = 0.014) was primarily evident in the IVW random‐effects model results, indicating a negative causal relationship. However, the effect on the HER2‐enriched subtype (OR = 0.84, CI = 0.73–0.96, *p* = 0.010) was primarily revealed in the IVW fixed‐effects model results, which also indicated a negative causal relationship. Both the MR‐Egger and WM methods for overall breast cancer and the luminal A subtype were consistent with the IVW results (*p* < 0.05). Regarding the HER2‐enriched subtype, even though the MR‐Egger analysis (OR = 0.83, CI = 0.66–1.04, *p* = 0.112) did not identify a statistically significant negative effect, its alignment with the direction of the results from the IVW and WM methods is noteworthy. However, the luminal B, luminal B HER2‐negative, and triple‐negative subtypes showed no significant effects in either the fixed‐ or random‐effects IVW models. The MR‐Egger analysis revealed a negative causal relationship for the luminal B HER2‐negative and triple‐negative subtypes. The magnitude of the effects of circulating triglycerides on the risk of different types of breast cancer is shown in Data S4.

### 3.3 Circulating Triglycerides and Survival Risk in Patients With Breast Cancer

**FIGURE 3:**
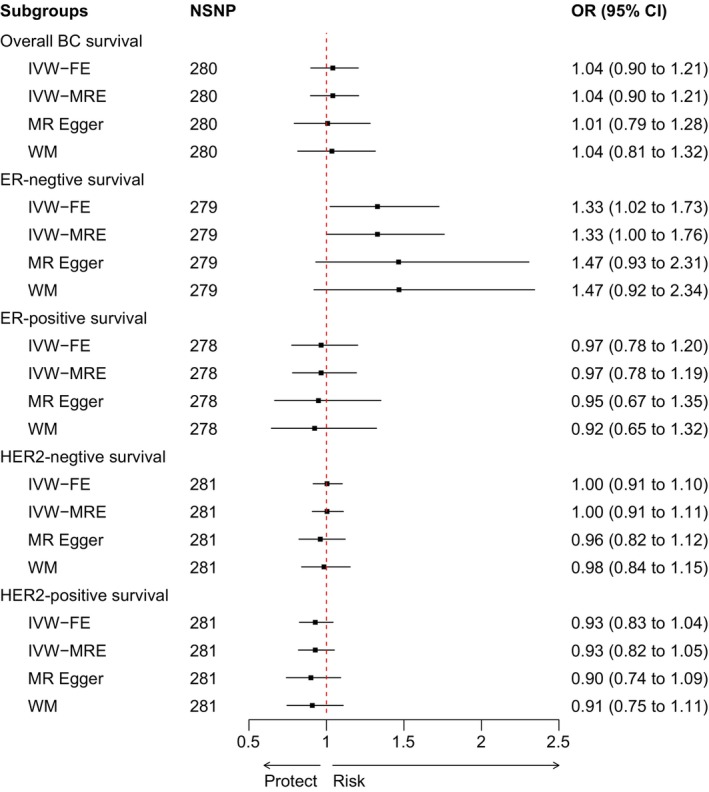
Forest plot of Mendelian randomization causal relationship between triglyceride levels and breast cancer survival risk. BC, breast cancer; CI, confidence interval; ER, estrogen receptor; FE, fixed‐effects model; HER2, human epidermal growth factor receptor 2; IVW, inverse‐variance weighted; MR, Mendelian Randomization; MRE, random‐effects model; NSNP, number of single‐nucleotide polymorphism; OR, odds ratio; WM, weighted median.

In the investigation of the causal relationship between circulating triglycerides and the prognosis of ER‐negative breast cancer (Figure 3), both the fixed‐effects (OR = 1.33, CI = 1.02–1.73, *p* = 0.033) and random‐effects (OR = 1.33, CI = 1.00–1.76, *p* = 0.047) IVW models revealed that circulating triglycerides are a risk factor for the prognosis of ER‐negative breast cancer. Although the results of the MR‐Egger (OR = 1.47, CI = 0.93–2.31, *p* = 0.099) and WM (OR = 1.47, CI = 0.92–2.34, *p* = 0.107) analyses did not reach statistical significance, the results followed the same trend as the IVW results. However, for overall breast cancer, ER‐positive breast cancer, HER2‐positive breast cancer, and HER2‐negative breast cancer survival prognosis, no statistically significant causal relationships were observed through any of the four MR methods. Data S5 shows the magnitude of the causal effects.

### 3.4 Sensitivity Analysis

In the MR‐Egger intercept test results, no indication of directional pleiotropy was observed in the relationship between triglyceride levels and the risk of overall (intercept = 0.002172842, *p* = 0.051), luminal A (intercept = 0.002425546, *p* = 0.079), or HER2‐enriched (intercept = 0.000402602, *p* = 0.903) breast cancer. Similarly, no evidence of directional pleiotropy was observed in the MR‐Egger (intercept = −0.003483606, *p* = 0.591) survival causal relationship test for ER‐negative breast cancer survival prognosis risk, further reinforcing the stability of the aforementioned results. No obvious asymmetry was observed in the funnel plot (Data S6 and S7). The results of the leave‐one‐out analysis validated that the exclusion of a single SNP had very little impact on the aforementioned causal relationships, again confirming the stability of our results (Data S8 and S9). Finally, after further identification and removal of SNPs with potential horizontal pleiotropy using Mendelian Randomization Pleiotropy RESidual Sum and Outlier testing and repeating the MR analysis, the results remained consistent with the previous findings (Data S10).

## 4 Discussion

We conducted the largest study thus far to examine the causal link between circulating triglycerides and the prevalence of the five molecular subtypes of breast cancer. Moreover, we evaluated the effect of circulating triglycerides on the survival of patients with the ER and HER2 breast cancer subtypes. Based on our findings, circulating triglycerides are protective factors against the occurrence of overall breast cancer, HER2 (overexpressing type) breast cancer, and luminal A breast cancer, but they have no significant impact on the occurrence of the other molecular subtypes of breast cancer. Meanwhile, circulating triglycerides are a risk factor for the survival of patients with ER‐negative breast cancer, but they have no significant impact on the survival of patients with the other molecular subtypes of breast cancer distinguished by ER and HER2. Sensitivity analysis confirmed that these results were not affected by horizontal pleiotropy. The findings of the present study are consistent with those of previous studies [44, 45]. Our findings indicate that triglyceride concentrations in peripheral blood may have different biological mechanisms and functions in individuals who have versus those who have not developed breast cancer.

Previous studies on the relationship between obesity and breast cancer risk have reported inconsistent results [19]. A meta‐analysis of 4570 patients with postmenopausal breast cancer revealed that an increase of 5 kg in body weight was associated with an approximately 11% increase in the risk of postmenopausal breast cancer. However, no linear relationship was observed between obesity and premenopausal breast cancer in a study involving 2409 cases of premenopausal breast cancer [46]. In addition, a review conducted by the World Cancer Research Fund International based on 119 studies, which included over 260,000 breast cancer cases, revealed that obesity might reduce the risk of breast cancer in younger women [47]. Similar controversies surround the influence of obesity and triglycerides on the survival of patients with breast cancer [48, 49]. Fassio [50] et al. found that vitamin D deficiency occurred in 60% of subjects who did not receive vitamin D supplementation within 1 year of breast cancer diagnosis and that vitamin D deficiency was associated with the development of hypertriglyceridemia and a poor prognosis for breast cancer. In contrast, the findings of a case–control study with a prospective meta‐analysis did not indicate that hypertriglyceridemia was associated with breast cancer development [51, 52]. The results of the study by Mad et al. [53] suggest that it is important to monitor lipid levels throughout the treatment of breast cancer patients, especially during neo‐adjuvant chemotherapy, where the presence of high triglycerides predicts a better prognosis compared to hypercholesterolemia.

This could be related to the sample size, confounding factors, or heterogeneity of breast cancer. Notably, these analyses only indicated correlations and did not reveal causal relationships. Triglycerides, which are important indicators of lipid metabolism, are also a main indicator of obesity [54]. In addition to serving as a basic component of cellular structures and participating in basic physiological functions, such as energy storage and metabolism, triglycerides play a significant role in the initiation and progression of tumors. They participate in the regulation of gene expression and signaling pathways and regulate downstream cell functions [55].

Polyunsaturated fatty acids stored in triglycerides may inhibit the inflammatory response in breast tissues by regulating cyclooxygenase 2 expression, thereby reducing the breast cancer risk [56]. Polyunsaturated fatty acids can also affect HER2 levels and activation, thereby interfering with the lipid microdomains (lipid rafts) of the cell membrane and reducing the signal transduction of oncogenic proteins and cell proliferation [57]. This may explain why triglycerides reduce the risk of the luminal A and HER2‐enriched breast cancer subtypes. However, adipokines such as leptin, plasminogen activator inhibitor‐1, and proinflammatory molecules produced during fat metabolism, such as tumor necrosis factor‐α and interleukin‐6, may also play certain carcinogenic roles [58]. Studies have shown that cancer cells use lipid metabolism to regulate the activity of the matrix and immune cells to gain an advantage, resist treatment, and promote recurrence [59]. The specific molecular mechanisms linking triglyceride levels and breast cancer remain unclear, and further experimental research and clinical studies are required to explore these potential molecular mechanisms.

In a previous study, we observed that, under certain circumstances, some factors that are usually regarded as unhealthy may actually have a protective effect on the body [60]. However, we do not encourage the prevention of breast cancer through excessive fat intake, as obesity may increase the risk of diseases in multiple body systems, such as cardiovascular metabolism; digestive, respiratory, nervous, and musculoskeletal systems; and infectious diseases [61]. The findings of this study remind us that the complexity of biological systems far exceeds our imaginations. Many aspects of the relationship between triglycerides and breast cancer remain unknown, and we cannot simply categorize triglycerides as good or bad. Therefore, understanding the relationship between circulating triglycerides and breast cancer from the perspective of the heterogeneity of breast cancer molecular subtypes is crucial for promoting scientific progress.

This study had some limitations. We restricted the genetic background of the participants in the MR study to individuals of European descent to avoid any potential confusion caused by population distribution; therefore, our conclusions are not generalizable to individuals of other races. Although we made every effort to minimize the bias caused by confounding factors, we could not avoid the potential pleiotropy caused by unknown confounders. Because the analytical framework of two‐sample MR relies on linear assumptions, we did not explore and evaluate the nonlinear relationship between circulating triglycerides and breast cancer. The relationship between triglycerides and breast cancer incidence and survival may vary owing to menstrual states; however, because of the limitations of the existing data, we could not confirm the relationship between breast cancer and premenopausal and postmenopausal status.

Despite the inherent limitations of our study, it stands unparalleled in both scale and depth when probing the relationship between circulating triglycerides and the five molecular subtypes of breast cancer, delivering powerful and eye‐catching statistical evidence. These findings bolster the precision with which physicians can assess breast cancer risks and devise preventive strategies, offering a precious reference for clinical practices. Furthermore, our research highlights the intricate nature of circulating triglycerides, suggesting new avenues for exploration in relevant future studies. Through this endeavor, we aim to deepen the public's understanding of the multifaceted role of circulating triglycerides.

## 5 Conclusions

The impact of circulating triglycerides on the development and survival of patients with breast cancer could be contingent on the molecular subtype. We found that circulating triglycerides may have a protective effect against the occurrence of HER2‐overexpressing and luminal A breast cancer but may pose a risk to the survival of patients with ER‐negative breast cancer. The role of triglycerides in the etiology and survival of patients with breast cancer is likely more complex than anticipated. However, merely categorizing triglycerides as risk factors for breast cancer may be inadequate. These findings provide a new perspective to potentially unravel the biological mechanisms by which circulating triglycerides influence the heterogeneity of breast cancer, potentially leading to the discovery of new signaling pathways and potential therapeutic targets and promoting the development of precise medical strategies. In addition, our findings could help both the public and professionals establish scientifically accurate perceptions of health.

## Author Contributions

**Wu Zhang:** conceptualization (equal), formal analysis (equal), resources (equal), software (equal), writing – original draft (equal). **Zhiru Li:** investigation (equal), methodology (equal), validation (equal), visualization (equal), writing – original draft (equal). **Yuquan Huang:** methodology (equal), software (equal), visualization (equal), writing – original draft (equal). **Jing Zhao:** supervision (equal), writing – review and editing (equal). **Shaowei Guo:** investigation (equal), project administration (equal), writing – review and editing (equal). **Qian Wang:** data curation (equal), investigation (equal), writing – review and editing (equal). **Sihan Guo:** resources (equal), software (equal), writing – original draft (equal). **Qingxia Li:** funding acquisition (equal), methodology (equal), project administration (equal), supervision (equal), writing – review and editing (equal).

## Ethics Statement

This research has been conducted using published studies and consortia providing publicly available summary statistics. All original studies have been approved by the corresponding ethical review boards, and the participants have provided informed consent. In addition, no individual‐level data was used in this study; therefore, no new ethical review board approval was required.

## Consent

The authors have nothing to report.

## Conflicts of Interest

The authors declare no conflicts of interest.

## Supporting information


Data S1.

Data S2.

Data S3.

Data S4.

Data S5.

Data S6.

Data S7.

Data S8.

Data S9.

Data S10.

Data S11.

## Data Availability

The datasets analyzed during the current study can be found in the BCAC repository (https://bcac.ccge.medschl.cam.ac.uk/), the MRC‐IEU repository (https://gwas.mrcieu.ac.uk), and the FinnGen repository (https://r7.finngen.fi/).
