# Supplementary material for: Complex Role of Circulating Triglycerides in Breast Cancer Onset and Survival: Insights From Two‐Sample Mendelian Randomization Study
Source: Cancer Med. 2025 Feb 17;14(4):e70698. doi: 10.1002/cam4.70698 (PMC11831496; doi:10.1002/cam4.70698)
Supplement: Supplementary file 1 — Data S1. [file CAM4-14-e70698-s008.docx]

Additional file 1: Sample sizes for overall breast cancer, GWAS subtypes, and tumor markers: estrogen receptor (negative, positive, and unknown), progesterone receptor (negative, positive, and unknown), HER2 (negative, positive, and unknown), and grade (1, 2, 3, and unknown)^[[1]](#endnote-1)^

| Subtypes of Breast Cancer: GWAS Sample Sizes | | |
| --- | --- | --- |
|  | Cases | Controls |
| Subtypes | 106278 | 91477 |
| Tumor Marker Subtypes: GWAS Sample Sizes | | |
| ER Status | Negative | 16883 |
|  | Positive | 69963 |
|  | Unknown | 19432 |
| PR Status | Negative | 24260 |
|  | Positive | 51546 |
|  | Unknown | 30472 |
| HER2 Status | Negative | 47645 |
|  | Positive | 9502 |
|  | Unknown | 49131 |
| Grade Status | 1 | 15566 |
|  | 2 | 37532 |
|  | 3 | 24360 |
|  | Unknown | 28820 |
| Breast Cancer Subtypes Description | | |
| Luminal A | ER+ and/or PR+, HER2-, grade 1 and 2 | |
| Luminal B | ER+ and/or PR+, HER2+ | |
| Luminal B and Her2 negative | ER+ and/or PR+, HER2-, grade 3 | |
| HER2 + | ER-, PR-, HER2+ | |
| Triple - | ER-, PR-, HER2- | |

GWAS, genome-wide association study; ER, estrogen receptor; PR, progesterone receptor.

1. Peruchet-Noray L, Dimou N, Sedlmeier AM, et al. Body Shape Phenotypes and Breast Cancer Risk: A Mendelian Randomization Analysis. Cancers (Basel). 2023;15(4):1296. Published 2023 Feb 17. doi:10.3390/cancers15041296 [↑](#endnote-ref-1)
