# Supplementary material for: Complex Role of Circulating Triglycerides in Breast Cancer Onset and Survival: Insights From Two‐Sample Mendelian Randomization Study
Source: Cancer Med. 2025 Feb 17;14(4):e70698. doi: 10.1002/cam4.70698 (PMC11831496; doi:10.1002/cam4.70698)
Supplement: Supplementary file 2 — Data S2. [file CAM4-14-e70698-s006.docx]

| SNP | effect_  allele | other_  allele | eaf | beta | se | p |
| --- | --- | --- | --- | --- | --- | --- |
| rs880315 | C | T | 0.340211 | -0.011772 | 0.002105 | 2.20E-08 |
| rs2811964 | G | A | 0.906954 | 0.0196101 | 0.0034488 | 1.30E-08 |
| rs11416534 | C | G | 0.023157 | 0.0818126 | 0.0066359 | 6.30E-35 |
| rs61780049 | G | A | 0.14886 | 0.0153212 | 0.0027928 | 4.10E-08 |
| rs11206374 | A | G | 0.224815 | 0.0250232 | 0.0023758 | 6.10E-26 |
| rs2131311 | G | A | 0.714636 | -0.012418 | 0.0022275 | 2.50E-08 |
| rs72904737 | A | G | 0.087416 | -0.027145 | 0.003518 | 1.20E-14 |
| rs213494 | T | C | 0.648345 | 0.0156106 | 0.0020758 | 5.50E-14 |
| rs74090351 | A | G | 0.068143 | -0.02501 | 0.0039366 | 2.10E-10 |
| rs626787 | G | C | 0.352889 | -0.077827 | 0.0020809 | 1.00E-200 |
| rs698927 | C | A | 0.183785 | -0.018306 | 0.002563 | 9.20E-13 |
| rs1938566 | T | C | 0.834537 | -0.021213 | 0.0026705 | 2.00E-15 |
| rs61785481 | A | G | 0.411762 | 0.0111206 | 0.0020225 | 3.80E-08 |
| rs320369 | G | A | 0.683292 | -0.012533 | 0.0021457 | 5.20E-09 |
| rs1044808 | C | G | 0.081237 | -0.024749 | 0.0036219 | 8.30E-12 |
| rs1760801 | A | G | 0.295768 | -0.020298 | 0.0021844 | 1.50E-20 |
| rs9425589 | A | G | 0.566781 | -0.013762 | 0.0020018 | 6.20E-12 |
| rs6700266 | A | G | 0.342465 | -0.01245 | 0.0020932 | 2.70E-09 |
| rs36043408 | A | G | 0.502153 | -0.012807 | 0.0019855 | 1.10E-10 |
| rs11240358 | A | G | 0.394026 | 0.0135651 | 0.0020295 | 2.30E-11 |
| rs11118310 | T | A | 0.593476 | 0.0193178 | 0.0020201 | 1.10E-21 |
| rs61830291 | C | A | 0.095791 | 0.0287484 | 0.0033791 | 1.80E-17 |
| rs11122450 | G | T | 0.611733 | -0.048175 | 0.0020373 | 1.30E-123 |
| rs1043897 | T | G | 0.415585 | -0.014664 | 0.0020152 | 3.40E-13 |
| rs3820897 | C | T | 0.819861 | 0.0197269 | 0.002603 | 3.50E-14 |
| rs6531216 | A | G | 0.530956 | 0.0182469 | 0.0019859 | 4.00E-20 |
| rs676210 | A | G | 0.205293 | -0.073517 | 0.0024497 | 7.10E-198 |
| rs1659711 | A | C | 0.502479 | 0.0151799 | 0.0019818 | 1.90E-14 |
| rs3739095 | A | G | 0.436474 | -0.061838 | 0.0019971 | 1.00E-200 |
| rs2141371 | A | G | 0.695893 | 0.0468845 | 0.0022127 | 1.20E-99 |
| rs7561670 | A | G | 0.197315 | 0.0145384 | 0.0024906 | 5.30E-09 |
| rs17326656 | T | G | 0.238472 | 0.0174394 | 0.0023337 | 7.80E-14 |
| rs1520456 | T | A | 0.443064 | 0.0109572 | 0.0020037 | 4.50E-08 |
| rs7424120 | T | C | 0.601724 | -0.012356 | 0.0020307 | 1.20E-09 |
| rs1009360 | C | T | 0.41859 | -0.018483 | 0.0020079 | 3.40E-20 |
| rs10172544 | A | C | 0.408707 | -0.011229 | 0.0020155 | 2.50E-08 |
| rs11904650 | G | A | 0.020999 | 0.0410641 | 0.0069242 | 3.00E-09 |
| rs954244 | G | C | 0.254641 | 0.015323 | 0.0022742 | 1.60E-11 |
| rs4662414 | G | A | 0.446608 | -0.012057 | 0.0019918 | 1.40E-09 |
| rs6432622 | G | A | 0.490264 | -0.010887 | 0.0019801 | 3.80E-08 |
| rs4128205 | C | A | 0.509173 | 0.0115011 | 0.0019947 | 8.10E-09 |
| rs13389219 | T | C | 0.392551 | -0.037624 | 0.002027 | 6.60E-77 |
| rs1420384 | T | G | 0.66699 | -0.01279 | 0.0021053 | 1.20E-09 |
| rs3731696 | G | A | 0.121188 | 0.0218939 | 0.0030318 | 5.10E-13 |
| rs2382825 | T | C | 0.622954 | -0.013477 | 0.0020492 | 4.80E-11 |
| rs78058190 | A | G | 0.05045 | 0.0816071 | 0.0051065 | 1.70E-57 |
| rs2943645 | T | C | 0.646594 | 0.0402918 | 0.0020715 | 2.90E-84 |
| rs12475332 | G | T | 0.260988 | -0.014001 | 0.0022539 | 5.20E-10 |
| rs4675812 | A | G | 0.587882 | -0.014231 | 0.0020118 | 1.50E-12 |
| rs6798755 | T | C | 0.065529 | -0.025102 | 0.0040186 | 4.20E-10 |

| rs3103310 | G | A | 0.241785 | 0.0202549 | 0.0023665 | 1.10E-17 |
| --- | --- | --- | --- | --- | --- | --- |
| rs6792725 | G | A | 0.692404 | -0.015254 | 0.0022189 | 6.20E-12 |
| rs9831084 | C | T | 0.461922 | -0.011852 | 0.0019966 | 2.90E-09 |
| rs2276853 | A | G | 0.59607 | 0.0111258 | 0.0020231 | 3.80E-08 |
| rs6800707 | G | C | 0.810543 | 0.0298848 | 0.0025383 | 5.30E-32 |
| rs6805924 | T | G | 0.430548 | 0.0109684 | 0.0020057 | 4.50E-08 |
| rs684773 | C | A | 0.766797 | 0.0291133 | 0.0023472 | 2.50E-35 |
| rs73238173 | G | C | 0.129955 | -0.016594 | 0.0029543 | 1.90E-08 |
| rs62271373 | A | T | 0.059967 | 0.0419639 | 0.0042659 | 7.80E-23 |
| rs62274099 | T | C | 0.424205 | 0.0120882 | 0.0020242 | 2.30E-09 |
| rs10513688 | A | G | 0.097403 | 0.0247828 | 0.0033492 | 1.40E-13 |
| rs79287178 | A | G | 0.031151 | 0.0500946 | 0.0060044 | 7.20E-17 |
| rs2137557 | C | T | 0.645612 | 0.0117624 | 0.00208 | 1.60E-08 |
| rs11185542 | C | G | 0.727914 | -0.01264 | 0.0022385 | 1.60E-08 |
| rs13108218 | G | A | 0.614859 | -0.030517 | 0.0020581 | 9.70E-50 |
| rs2702544 | G | A | 0.658163 | 0.0116368 | 0.0020987 | 2.90E-08 |
| rs71603401 | G | A | 0.136804 | 0.0264991 | 0.0029175 | 1.10E-19 |
| rs73243877 | G | A | 0.168135 | 0.0288117 | 0.0026556 | 2.00E-27 |
| rs12504746 | T | C | 0.192857 | -0.015267 | 0.0025215 | 1.40E-09 |
| rs278981 | C | T | 0.758155 | 0.0125871 | 0.0022906 | 3.90E-08 |
| rs2237029 | A | G | 0.601353 | -0.01394 | 0.0020402 | 8.30E-12 |
| rs13101504 | G | T | 0.412851 | 0.0195598 | 0.0024075 | 4.50E-16 |
| rs11722924 | C | G | 0.53531 | 0.0129095 | 0.0019909 | 8.90E-11 |
| rs3775228 | T | C | 0.399717 | 0.0338197 | 0.002036 | 5.80E-62 |
| rs13118477 | A | G | 0.392654 | 0.0149897 | 0.0020385 | 1.90E-13 |
| rs6532798 | T | C | 0.697405 | 0.013792 | 0.0021635 | 1.80E-10 |
| rs13107325 | T | C | 0.074905 | 0.0300749 | 0.0037774 | 1.70E-15 |
| rs14882777 | G | A | 0.022544 | 0.0465516 | 0.0071064 | 5.70E-11 |
| rs1347188 | G | A | 0.245797 | 0.0139021 | 0.0023139 | 1.90E-09 |
| rs11100083 | C | T | 0.226214 | -0.016025 | 0.0023759 | 1.50E-11 |
| rs7735249 | G | C | 0.112767 | 0.0268635 | 0.00316 | 1.90E-17 |
| rs3936511 | G | A | 0.19191 | 0.0461274 | 0.0025216 | 9.50E-75 |
| rs192249 | G | C | 0.651188 | -0.013728 | 0.002089 | 5.00E-11 |
| rs37538 | C | G | 0.601553 | -0.014397 | 0.0020375 | 1.60E-12 |
| rs4976033 | G | A | 0.402122 | 0.0177909 | 0.0020518 | 4.30E-18 |
| rs1316753 | C | G | 0.394062 | -0.014573 | 0.0020297 | 7.00E-13 |
| rs7704653 | G | A | 0.723029 | 0.0157533 | 0.0022458 | 2.30E-12 |
| rs325485 | G | A | 0.602889 | -0.011744 | 0.0020404 | 8.60E-09 |
| rs7714361 | C | A | 0.233667 | 0.0138858 | 0.0023662 | 4.40E-09 |
| rs1045241 | T | C | 0.270943 | -0.020699 | 0.002247 | 3.20E-20 |
| rs193735 | A | G | 0.036665 | 0.0329807 | 0.0052992 | 4.90E-10 |
| rs72801474 | A | G | 0.091843 | -0.030794 | 0.0034415 | 3.60E-19 |
| rs970069 | T | C | 0.212039 | 0.0162322 | 0.0024346 | 2.60E-11 |
| rs7244 | A | G | 0.174166 | 0.0151978 | 0.0026181 | 6.40E-09 |
| rs6882076 | C | T | 0.634285 | 0.0331011 | 0.0020612 | 4.90E-58 |
| rs13354321 | C | T | 0.409787 | -0.015431 | 0.0020143 | 1.90E-14 |
| rs55646464 | T | G | 0.300087 | 0.0121913 | 0.0021702 | 1.90E-08 |
| rs62397245 | G | C | 0.22236 | 0.0149941 | 0.0023963 | 3.90E-10 |
| rs78588343 | A | G | 0.176216 | -0.015523 | 0.0026035 | 2.50E-09 |
| rs6924805 | T | G | 0.587637 | -0.011156 | 0.0020199 | 3.30E-08 |
| rs4134963 | T | C | 0.189878 | -0.018954 | 0.0025424 | 9.00E-14 |

| rs2275852 | G | T | 0.052332 | 0.025198 | 0.0044869 | 2.00E-08 |
| --- | --- | --- | --- | --- | --- | --- |
| rs28752924 | C | T | 0.445662 | 0.0265556 | 0.002057 | 4.00E-38 |
| rs2229092 | C | A | 0.062054 | 0.0256807 | 0.0041127 | 4.30E-10 |
| rs12661597 | T | G | 0.040154 | 0.0666295 | 0.0052625 | 9.70E-37 |
| rs28383314 | C | T | 0.624109 | 0.0379005 | 0.0020467 | 1.50E-76 |
| rs998584 | A | C | 0.482727 | 0.0401182 | 0.00199 | 2.20E-90 |
| rs729761 | G | T | 0.711859 | 0.0177868 | 0.0022144 | 9.50E-16 |
| rs13819177 | A | G | 0.017021 | -0.047172 | 0.0079188 | 2.60E-09 |
| rs2983896 | A | G | 0.21474 | 0.0137912 | 0.0024218 | 1.20E-08 |
| rs62427982 | T | C | 0.32217 | -0.013315 | 0.002132 | 4.20E-10 |
| rs9480889 | G | C | 0.783137 | 0.0163067 | 0.0024101 | 1.30E-11 |
| rs6916318 | T | A | 0.530533 | 0.0267283 | 0.0019907 | 4.20E-41 |
| rs19990490 | C | G | 0.320204 | -0.011845 | 0.0021301 | 2.70E-08 |
| rs17585887 | C | T | 0.591074 | -0.028585 | 0.0020166 | 1.30E-45 |
| rs9376511 | G | A | 0.203288 | -0.015499 | 0.0024651 | 3.20E-10 |
| rs73025562 | A | G | 0.245879 | 0.0138633 | 0.0023129 | 2.00E-09 |
| rs77009508 | G | A | 0.073734 | 0.0450912 | 0.0038 | 1.80E-32 |
| rs18669626 | T | C | 0.014693 | -0.104318 | 0.0082852 | 2.40E-36 |
| rs1835346 | G | A | 0.023874 | -0.039188 | 0.0065466 | 2.20E-09 |
| rs71538127 | G | C | 0.122052 | 0.0176494 | 0.0030372 | 6.20E-09 |
| rs852388 | C | G | 0.211278 | 0.0157124 | 0.002456 | 1.60E-10 |
| rs38189 | T | A | 0.634401 | -0.015316 | 0.0020629 | 1.10E-13 |
| rs10277582 | T | C | 0.118591 | -0.017371 | 0.0030802 | 1.70E-08 |
| rs2691553 | A | G | 0.51063 | 0.0140801 | 0.001986 | 1.30E-12 |
| rs7786102 | A | G | 0.715703 | -0.02837 | 0.0022009 | 5.10E-38 |
| rs1534696 | A | C | 0.541122 | -0.019766 | 0.0019913 | 3.20E-23 |
| rs11980456 | A | G | 0.291468 | 0.0123903 | 0.0022123 | 2.10E-08 |
| rs1799831 | T | C | 0.156029 | 0.024506 | 0.0027395 | 3.70E-19 |
| rs62459095 | T | C | 0.061781 | -0.031987 | 0.0042688 | 6.70E-14 |
| rs13229300 | T | C | 0.273078 | -0.013752 | 0.0022377 | 8.00E-10 |
| rs18409958 | C | T | 0.023168 | 0.0425191 | 0.0072309 | 4.10E-09 |
| rs42124 | A | G | 0.029175 | 0.074742 | 0.005859 | 2.90E-37 |
| rs71556711 | T | C | 0.089735 | -0.122109 | 0.0034706 | 1.00E-200 |
| rs14509945 | T | C | 0.040399 | -0.115425 | 0.0052174 | 1.90E-108 |
| rs12530679 | G | A | 0.48466 | -0.01213 | 0.0020168 | 1.80E-09 |
| rs41785 | A | C | 0.417487 | -0.015045 | 0.0020149 | 8.20E-14 |
| rs4731701 | T | C | 0.493088 | -0.03258 | 0.0019892 | 2.70E-60 |
| rs62473520 | C | T | 0.077998 | -0.0211 | 0.0038012 | 2.80E-08 |
| rs53524119 | G | A | 0.21449 | 0.0210699 | 0.0024599 | 1.10E-17 |
| rs7836833 | C | T | 0.320716 | 0.027108 | 0.0021146 | 1.30E-37 |
| rs7386288 | C | T | 0.744712 | -0.022855 | 0.0022755 | 9.70E-24 |
| rs1495741 | A | G | 0.779419 | -0.037591 | 0.0023819 | 4.20E-56 |
| rs78114419 | T | C | 0.053691 | -0.026878 | 0.004478 | 1.90E-09 |
| rs1388942 | G | A | 0.092936 | -0.028077 | 0.0034097 | 1.80E-16 |
| rs73597688 | A | C | 0.18651 | -0.078722 | 0.0025432 | 1.00E-200 |
| rs75591007 | G | T | 0.017765 | 0.161226 | 0.0074903 | 9.20E-103 |
| rs2407278 | G | A | 0.030154 | -0.033945 | 0.0058196 | 5.40E-09 |
| rs2081687 | C | T | 0.663201 | -0.026138 | 0.0020913 | 7.60E-36 |
| rs28439112 | A | T | 0.255528 | 0.0126014 | 0.0022675 | 2.70E-08 |
| rs13269725 | G | A | 0.078416 | 0.0349878 | 0.0036804 | 2.00E-21 |
| rs13265978 | C | T | 0.082718 | 0.0213535 | 0.0035865 | 2.60E-09 |

| rs6999569 | G | A | 0.470737 | -0.086089 | 0.0019806 | 1.00E-200 |
| --- | --- | --- | --- | --- | --- | --- |
| rs12546096 | G | A | 0.252369 | 0.0278972 | 0.0023098 | 1.40E-33 |
| rs72691637 | A | G | 0.187053 | -0.015426 | 0.0025658 | 1.80E-09 |
| rs1567353 | G | C | 0.307644 | 0.0148202 | 0.0021636 | 7.40E-12 |
| rs7847285 | C | T | 0.589213 | -0.01161 | 0.0020263 | 1.00E-08 |
| rs581080 | C | G | 0.819008 | 0.0175432 | 0.0025844 | 1.10E-11 |
| rs10811662 | A | G | 0.173212 | -0.01541 | 0.0026291 | 4.60E-09 |
| rs696825 | T | C | 0.252926 | -0.020284 | 0.0022876 | 7.50E-19 |
| rs10797119 | C | T | 0.536622 | 0.0157113 | 0.0020047 | 4.60E-15 |
| rs2131919 | G | A | 0.164266 | 0.0173511 | 0.002684 | 1.00E-10 |
| rs2244278 | A | C | 0.120553 | -0.026991 | 0.0030613 | 1.20E-18 |
| rs7861679 | T | C | 0.696815 | 0.0122025 | 0.0021674 | 1.80E-08 |
| rs4962112 | C | T | 0.528394 | -0.011042 | 0.0020048 | 3.60E-08 |
| rs2519093 | T | C | 0.184548 | -0.021158 | 0.0025619 | 1.50E-16 |
| rs4382584 | A | G | 0.274334 | 0.0129432 | 0.0022354 | 7.00E-09 |
| rs14010729 | G | A | 0.154707 | -0.0227 | 0.002753 | 1.60E-16 |
| rs3758413 | C | T | 0.417724 | 0.0111232 | 0.0020177 | 3.50E-08 |
| rs80276949 | A | G | 0.022677 | 0.0457563 | 0.0066901 | 8.00E-12 |
| rs55767272 | C | A | 0.065077 | -0.028201 | 0.004036 | 2.80E-12 |
| rs10822163 | G | C | 0.473369 | -0.032167 | 0.0019906 | 9.70E-59 |
| rs11000468 | T | C | 0.255269 | -0.014854 | 0.0023175 | 1.50E-10 |
| rs7077812 | C | T | 0.194911 | 0.0142284 | 0.0025061 | 1.40E-08 |
| rs11187019 | G | A | 0.550621 | -0.011644 | 0.0020055 | 6.40E-09 |
| rs2068888 | A | G | 0.450732 | -0.031822 | 0.0019959 | 3.20E-57 |
| rs11334442 | A | G | 0.060177 | 0.0434468 | 0.0042893 | 4.10E-24 |
| rs10883026 | T | C | 0.521569 | -0.01446 | 0.0020047 | 5.50E-13 |
| rs2487294 | T | G | 0.723477 | 0.0183191 | 0.0022208 | 1.60E-16 |
| rs56397607 | G | A | 0.182538 | 0.0174851 | 0.0025761 | 1.10E-11 |
| rs2773469 | G | A | 0.733286 | -0.018872 | 0.0022543 | 5.70E-17 |
| rs1133400 | G | A | 0.219923 | 0.0137462 | 0.0023976 | 9.80E-09 |
| rs11729124 | T | C | 0.036536 | 0.0297851 | 0.0052823 | 1.70E-08 |
| rs61885960 | A | T | 0.054205 | -0.031394 | 0.0044298 | 1.40E-12 |
| rs11728723 | A | G | 0.028185 | -0.039432 | 0.0061468 | 1.40E-10 |
| rs75268115 | G | A | 0.084699 | -0.020744 | 0.0035798 | 6.80E-09 |
| rs499293 | A | G | 0.65807 | -0.011791 | 0.002089 | 1.70E-08 |
| rs326222 | C | T | 0.697957 | 0.0252203 | 0.002157 | 1.40E-31 |
| rs3974807 | T | C | 0.189267 | 0.0159776 | 0.0025313 | 2.80E-10 |
| rs174566 | G | A | 0.34974 | 0.0485019 | 0.0020798 | 2.80E-120 |
| rs2302263 | T | C | 0.088863 | 0.0436112 | 0.0034905 | 8.00E-36 |
| rs10750766 | A | C | 0.709959 | 0.0193855 | 0.0021874 | 7.80E-19 |
| rs551243 | C | G | 0.469258 | 0.0143952 | 0.0019918 | 4.90E-13 |
| rs10899490 | T | C | 0.161369 | -0.017008 | 0.0026935 | 2.70E-10 |
| rs495033 | T | C | 0.927077 | -0.038768 | 0.0038085 | 2.40E-24 |
| rs513533 | G | A | 0.103811 | 0.116307 | 0.0033 | 1.00E-200 |
| rs61905078 | C | A | 0.073841 | 0.199898 | 0.0037949 | 1.00E-200 |
| rs15042365 | T | G | 0.006675 | 0.285824 | 0.0122924 | 1.40E-119 |
| rs7930797 | G | A | 0.494948 | 0.0267852 | 0.0023462 | 3.50E-30 |
| rs11723310 | A | G | 0.015176 | -0.073232 | 0.0086122 | 1.80E-17 |
| rs7308584 | A | G | 0.184436 | 0.0149992 | 0.0025691 | 5.30E-09 |
| rs7134375 | A | C | 0.431174 | -0.017144 | 0.002005 | 1.20E-17 |
| rs67981690 | G | A | 0.129887 | 0.0299225 | 0.0029745 | 8.30E-24 |

| rs7135509 | C | T | 0.293196 | -0.012134 | 0.002212 | 4.10E-08 |
| --- | --- | --- | --- | --- | --- | --- |
| rs13938698 | T | C | 0.092129 | -0.02169 | 0.0035608 | 1.10E-09 |
| rs35763453 | C | T | 0.058647 | 0.02845 | 0.0043514 | 6.20E-11 |
| rs4760254 | C | G | 0.23908 | -0.028141 | 0.0023249 | 1.00E-33 |
| rs75942983 | T | A | 0.083627 | -0.02013 | 0.0036121 | 2.50E-08 |
| rs4761234 | C | T | 0.484365 | -0.014024 | 0.0019938 | 2.00E-12 |
| rs12424054 | A | G | 0.232437 | 0.0191212 | 0.0023535 | 4.50E-16 |
| rs10773000 | T | G | 0.332276 | -0.014929 | 0.0021148 | 1.70E-12 |
| rs4765148 | T | G | 0.312686 | -0.025154 | 0.0021456 | 9.70E-32 |
| rs10773049 | C | T | 0.39535 | -0.02904 | 0.0020369 | 4.10E-46 |
| rs1340819 | C | A | 0.345011 | -0.012166 | 0.0020947 | 6.30E-09 |
| rs14977805 | C | A | 0.32832 | -0.015757 | 0.0022259 | 1.50E-12 |
| rs2812208 | C | G | 0.02089 | -0.048426 | 0.0069632 | 3.50E-12 |
| rs6562773 | G | A | 0.547533 | -0.012054 | 0.0020106 | 2.00E-09 |
| rs9561643 | C | A | 0.314726 | 0.0167149 | 0.0021448 | 6.50E-15 |
| rs9584870 | C | T | 0.366179 | -0.012356 | 0.0021038 | 4.30E-09 |
| rs7400002 | G | A | 0.230609 | 0.0139701 | 0.0023666 | 3.60E-09 |
| rs7140110 | C | T | 0.298338 | 0.0283172 | 0.0021781 | 1.20E-38 |
| rs79192570 | A | G | 0.14416 | -0.025184 | 0.0028361 | 6.70E-19 |
| rs56902258 | A | T | 0.196198 | -0.015143 | 0.0025129 | 1.70E-09 |
| rs2070341 | T | C | 0.60309 | 0.0113012 | 0.0020341 | 2.80E-08 |
| rs6572807 | G | A | 0.267065 | 0.0124759 | 0.002249 | 2.90E-08 |
| rs12880341 | C | T | 0.159023 | 0.0209696 | 0.0027365 | 1.80E-14 |
| rs2240533 | C | T | 0.309898 | -0.01299 | 0.0021523 | 1.60E-09 |
| rs61993685 | C | T | 0.076413 | -0.023409 | 0.00374 | 3.90E-10 |
| rs275184 | G | T | 0.162051 | -0.017349 | 0.0027526 | 2.90E-10 |
| rs11637681 | G | A | 0.276291 | 0.0125866 | 0.0022425 | 2.00E-08 |
| rs12902047 | C | A | 0.313907 | -0.012925 | 0.0021453 | 1.70E-09 |
| rs13997467 | C | T | 0.026131 | 0.143073 | 0.0062387 | 2.20E-116 |
| rs15046058 | C | T | 0.044425 | 0.0326952 | 0.0048497 | 1.60E-11 |
| rs2043085 | C | T | 0.612184 | -0.030803 | 0.0020416 | 1.90E-51 |
| rs1077835 | G | A | 0.220459 | 0.0473668 | 0.0024045 | 2.20E-86 |
| rs12440800 | T | A | 0.255149 | 0.0161 | 0.0022929 | 2.20E-12 |
| rs17184382 | C | A | 0.425367 | -0.021925 | 0.0020104 | 1.10E-27 |
| rs10152471 | A | G | 0.388656 | -0.013521 | 0.0020478 | 4.00E-11 |
| rs1037117 | A | G | 0.254798 | 0.0172213 | 0.0022915 | 5.70E-14 |
| rs14307645 | A | G | 0.018647 | 0.0403999 | 0.0073536 | 3.90E-08 |
| rs742036 | A | G | 0.375259 | -0.014363 | 0.0020511 | 2.50E-12 |
| rs28577186 | A | G | 0.664571 | -0.016343 | 0.0021167 | 1.20E-14 |
| rs12928099 | A | C | 0.296442 | -0.028212 | 0.0021813 | 2.90E-38 |
| rs12446515 | T | C | 0.322889 | -0.033421 | 0.0021359 | 3.50E-55 |
| rs2937124 | T | C | 0.362769 | -0.018256 | 0.0021234 | 8.10E-18 |
| rs34682685 | A | G | 0.104198 | 0.0337428 | 0.0032699 | 5.80E-25 |
| rs2925979 | C | T | 0.699976 | -0.032209 | 0.002171 | 8.50E-50 |
| rs4471666 | G | T | 0.069488 | -0.022461 | 0.003967 | 1.50E-08 |
| rs12926107 | G | A | 0.454903 | 0.0126364 | 0.0020014 | 2.70E-10 |
| rs12948505 | T | C | 0.194119 | 0.0138887 | 0.0025121 | 3.20E-08 |
| rs11078597 | C | T | 0.186613 | 0.0191708 | 0.0025513 | 5.70E-14 |
| rs2304969 | T | G | 0.144932 | -0.016147 | 0.0028501 | 1.50E-08 |
| rs7215055 | G | A | 0.062892 | 0.0389233 | 0.0041006 | 2.30E-21 |
| rs11238190 | T | A | 0.06731 | 0.0231649 | 0.0040086 | 7.50E-09 |

| rs11225926 | A | C | 0.028291 | 0.139849 | 0.0060137 | 1.30E-119 |
| --- | --- | --- | --- | --- | --- | --- |
| rs45445495 | A | C | 0.049449 | 0.0289415 | 0.0047475 | 1.10E-09 |
| rs10775406 | G | A | 0.759694 | 0.0206571 | 0.0023258 | 6.60E-19 |
| rs12185242 | C | A | 0.454836 | 0.0175265 | 0.001999 | 1.80E-18 |
| rs1292065 | G | C | 0.709244 | -0.013924 | 0.0021867 | 1.90E-10 |
| rs1801689 | C | A | 0.030543 | -0.06613 | 0.0057772 | 2.40E-30 |
| rs60856912 | T | G | 0.162906 | 0.0248329 | 0.0027083 | 4.80E-20 |
| rs9889402 | A | G | 0.728192 | 0.0122827 | 0.0022293 | 3.60E-08 |
| rs4969179 | G | T | 0.60445 | -0.017753 | 0.0020365 | 2.80E-18 |
| rs6506033 | T | C | 0.072523 | -0.022895 | 0.003839 | 2.50E-09 |
| rs11664106 | T | A | 0.374102 | -0.012583 | 0.0021082 | 2.40E-09 |
| rs867939 | A | G | 0.57621 | -0.013605 | 0.0020252 | 1.80E-11 |
| rs7239575 | C | T | 0.490494 | -0.016077 | 0.0019913 | 6.80E-16 |
| rs921971 | C | T | 0.265901 | 0.01565 | 0.002256 | 4.00E-12 |
| rs2187114 | A | G | 0.101024 | -0.018515 | 0.0033038 | 2.10E-08 |
| rs4804101 | T | G | 0.439008 | 0.0193079 | 0.0020072 | 6.60E-22 |
| rs11878235 | A | G | 0.593373 | -0.012703 | 0.0020426 | 5.00E-10 |
| rs12891 | C | G | 0.431914 | -0.010981 | 0.0020148 | 5.00E-08 |
| rs11150053 | A | G | 0.056946 | -0.041901 | 0.004293 | 1.70E-22 |
| rs11684306 | A | G | 0.019327 | -0.226505 | 0.0072101 | 1.00E-200 |
| rs58542926 | T | C | 0.074608 | -0.103184 | 0.0037807 | 5.30E-164 |
| rs18824755 | T | C | 0.013117 | -0.134286 | 0.0091313 | 5.90E-49 |
| rs2081048 | C | T | 0.345256 | -0.013958 | 0.002104 | 3.30E-11 |
| rs62102718 | T | A | 0.286196 | 0.0202348 | 0.0022014 | 3.90E-20 |
| rs369599 | T | C | 0.305647 | -0.022575 | 0.0021631 | 1.70E-25 |
| rs11453601 | T | C | 0.026059 | 0.0769857 | 0.0062557 | 8.40E-35 |
| rs75627662 | T | C | 0.204887 | 0.0835132 | 0.0024615 | 1.00E-200 |
| rs11186803 | T | A | 0.193002 | -0.015689 | 0.0025894 | 1.40E-09 |
| rs12052128 | G | A | 0.657263 | -0.017931 | 0.0021015 | 1.40E-17 |
| rs12151142 | C | T | 0.444479 | 0.0162101 | 0.0020048 | 6.20E-16 |
| rs15123540 | T | C | 0.015827 | 0.0521833 | 0.0081514 | 1.50E-10 |
| rs14914283 | T | C | 0.156359 | 0.0169358 | 0.0027651 | 9.10E-10 |
| rs6028716 | A | G | 0.258428 | -0.012822 | 0.002283 | 2.00E-08 |
| rs2092203 | T | C | 0.481154 | 0.0136756 | 0.0019932 | 6.80E-12 |
| rs6073958 | C | T | 0.198674 | 0.0556952 | 0.0024934 | 1.60E-110 |
| rs55966194 | G | C | 0.281997 | -0.017851 | 0.0022125 | 7.10E-16 |
| rs7274718 | A | G | 0.598516 | 0.0159491 | 0.0020292 | 3.80E-15 |
| rs8126001 | T | C | 0.489576 | -0.016378 | 0.0019968 | 2.40E-16 |
| rs394872 | T | C | 0.536237 | 0.0111807 | 0.0020029 | 2.40E-08 |
| rs140288 | A | G | 0.566711 | -0.013262 | 0.0020084 | 4.00E-11 |
| rs134551 | T | C | 0.335438 | -0.011654 | 0.0021111 | 3.40E-08 |
| rs5755799 | G | C | 0.453747 | 0.0118859 | 0.0020034 | 3.00E-09 |
| rs2267373 | T | C | 0.580693 | 0.0215638 | 0.0020215 | 1.40E-26 |
| rs2071887 | A | T | 0.344804 | 0.016312 | 0.0020967 | 7.30E-15 |
| rs4253750 | C | T | 0.214455 | 0.0177576 | 0.002437 | 3.20E-13 |
