# Supplementary material for: Complex Role of Circulating Triglycerides in Breast Cancer Onset and Survival: Insights From Two‐Sample Mendelian Randomization Study
Source: Cancer Med. 2025 Feb 17;14(4):e70698. doi: 10.1002/cam4.70698 (PMC11831496; doi:10.1002/cam4.70698)
Supplement: Supplementary file 3 — Data S3. [file CAM4-14-e70698-s011.docx]

Additional file 3: Heterogeneity testing table for causal relationship between triglyceride levels and risk of different types of breast cancer and survival prognosis

| Outcome | Exposure | Method | Q | Q_df | Q_pval |
| --- | --- | --- | --- | --- | --- |
| allBC risk | Triglycerides | MR Egger | 601.200371 | 265 | 3.69E-28 |
|  | Triglycerides | Inverse variance weighted | 609.8958651 | 266 | 4.75E-29 |
| LuminalA Subtype-BC risk | Triglycerides | MR Egger | 533.5602151 | 264 | 2.12E-20 |
|  | Triglycerides | Inverse variance weighted | 539.8307052 | 265 | 6.07E-21 |
| LuminalB Subtype-BC risk | Triglycerides | MR Egger | 320.0703461 | 266 | 0.012861067 |
|  | Triglycerides | Inverse variance weighted | 320.0735315 | 267 | 0.014317796 |
| LuminalB HER2 negative Subtype-BC risk | Triglycerides | MR Egger | 372.0602593 | 266 | 1.84E-05 |
|  | Triglycerides | Inverse variance weighted | 381.339282 | 267 | 5.26E-06 |
| HER2 Subtype-BC risk | Triglycerides | MR Egger | 304.4895711 | 266 | 0.052294684 |
|  | Triglycerides | Inverse variance weighted | 304.5063101 | 267 | 0.056908505 |
| Triple negative Subtype-BC risk | Triglycerides | MR Egger | 327.2483638 | 266 | 0.006145587 |
|  | Triglycerides | Inverse variance weighted | 333.3594815 | 267 | 0.00355635 |
| all BC survival | Triglycerides | MR Egger | 287.6832671 | 278 | 0.332076957 |
|  | Triglycerides | Inverse variance weighted | 287.8009219 | 279 | 0.345728189 |
| ER+ survival | Triglycerides | MR Egger | 259.6846843 | 276 | 0.751772764 |
|  | Triglycerides | Inverse variance weighted | 259.7004829 | 277 | 0.764972793 |
| ER- survival | Triglycerides | MR Egger | 320.9907638 | 277 | 0.035437059 |
|  | Triglycerides | Inverse variance weighted | 321.3266562 | 278 | 0.037721046 |
| HER2+ survival | Triglycerides | MR Egger | 319.0021444 | 279 | 0.049824513 |
|  | Triglycerides | Inverse variance weighted | 319.1941672 | 280 | 0.053416582 |
| HER2- survival | Triglycerides | MR Egger | 318.2843202 | 279 | 0.052751891 |
|  | Triglycerides | Inverse variance weighted | 318.9103414 | 280 | 0.054622591 |
