# Supplementary figures and images for: Complex Role of Circulating Triglycerides in Breast Cancer Onset and Survival: Insights From Two‐Sample Mendelian Randomization Study
Source: Cancer Med. 2025 Feb 17;14(4):e70698. doi: 10.1002/cam4.70698 (PMC11831496; doi:10.1002/cam4.70698)

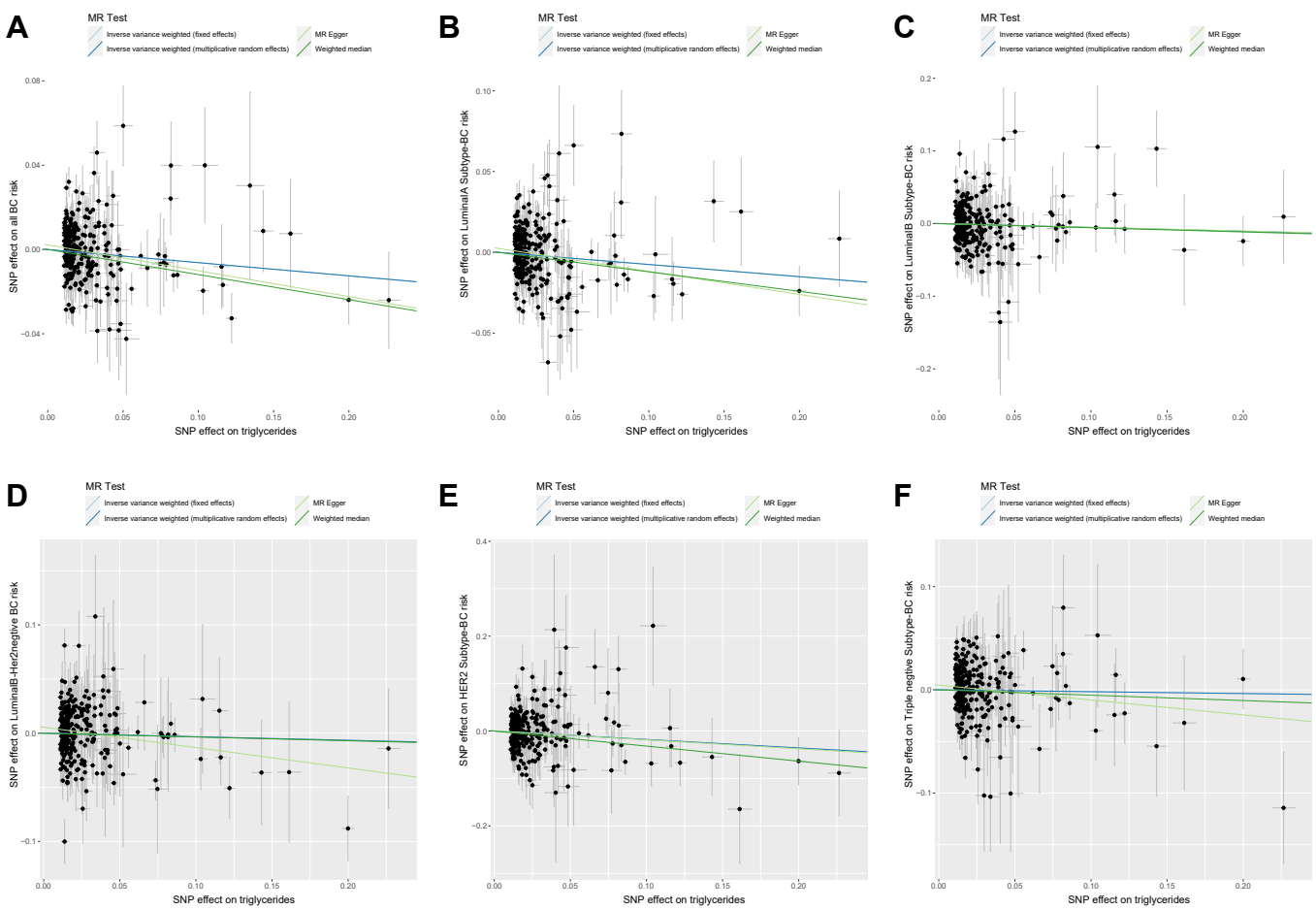

Supplement: Supplementary file 4 — Data S4. [file CAM4-14-e70698-s001.pdf]

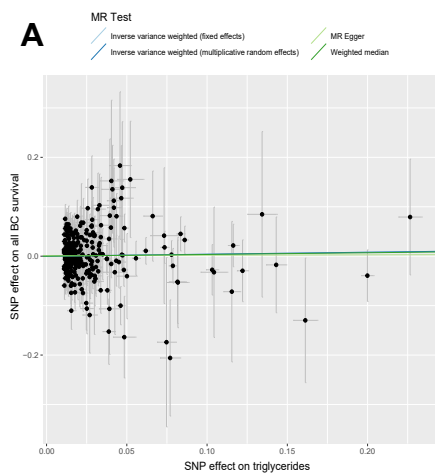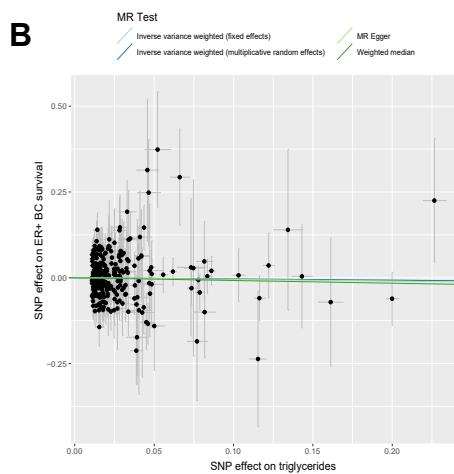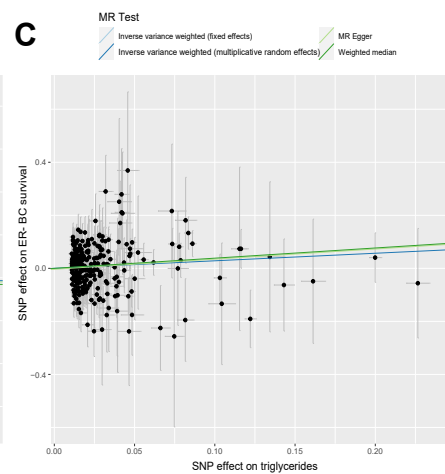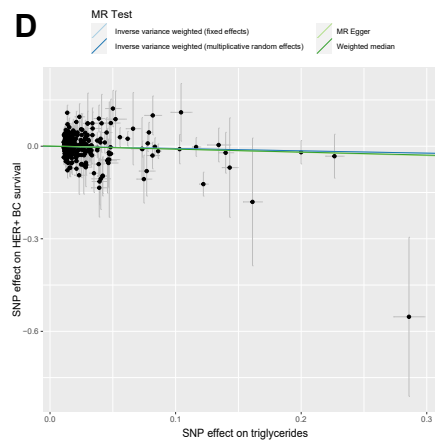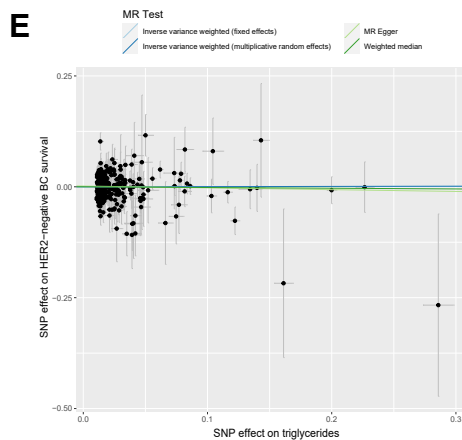

Supplement: Supplementary file 5 — Data S5. [file CAM4-14-e70698-s003.pdf]

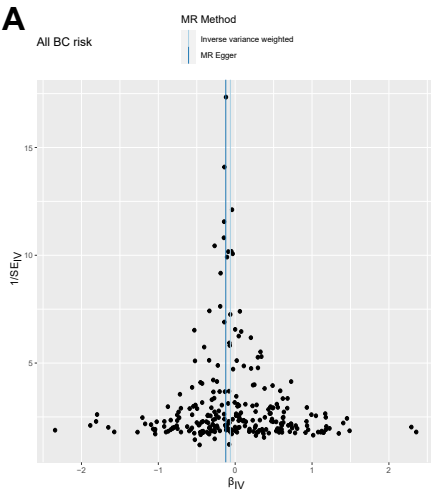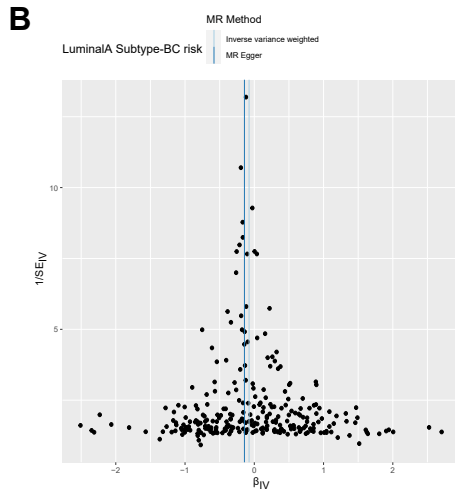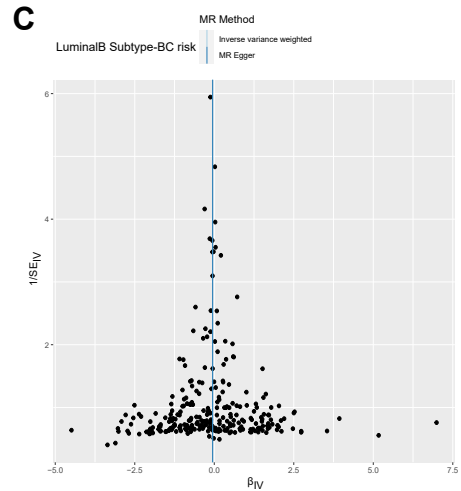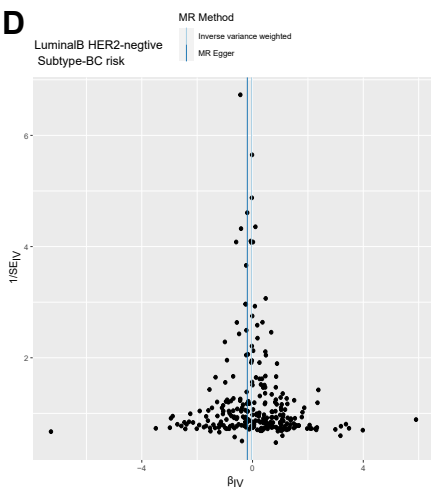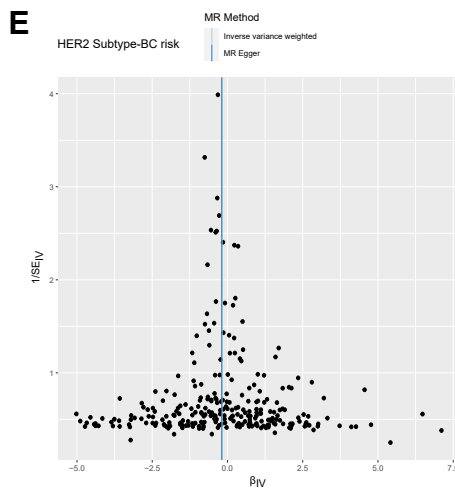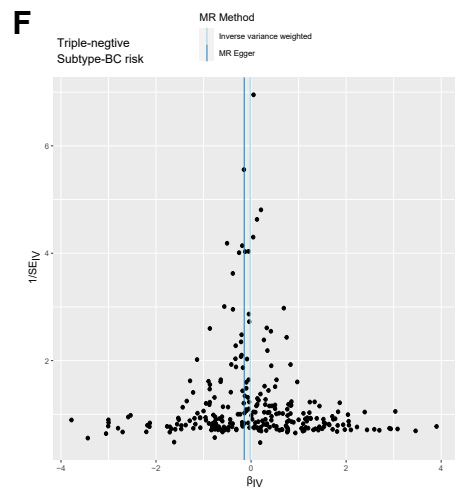

Supplement: Supplementary file 6 — Data S6. [file CAM4-14-e70698-s005.pdf]

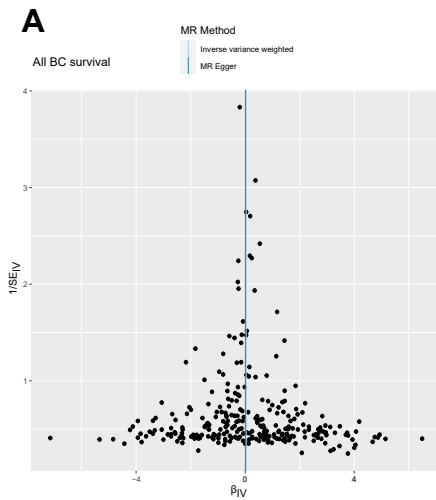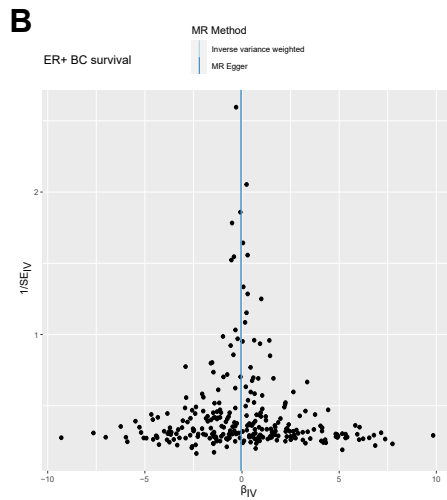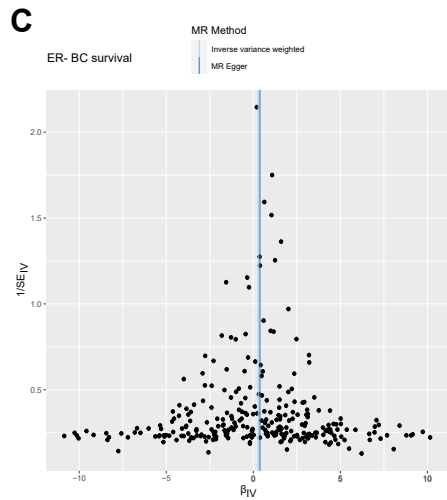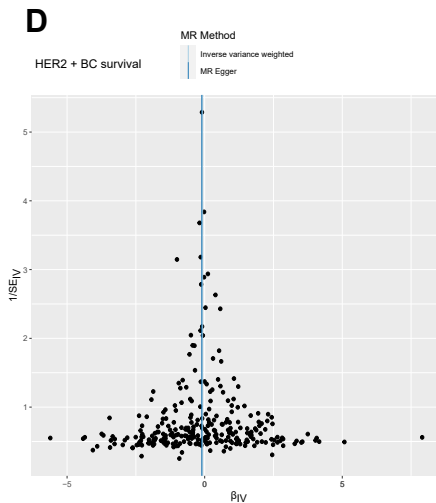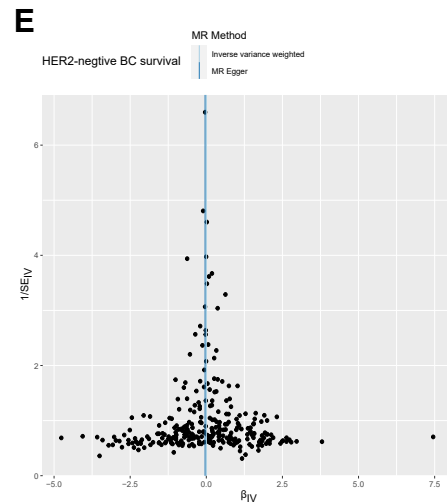

Supplement: Supplementary file 7 — Data S7. [file CAM4-14-e70698-s004.pdf]

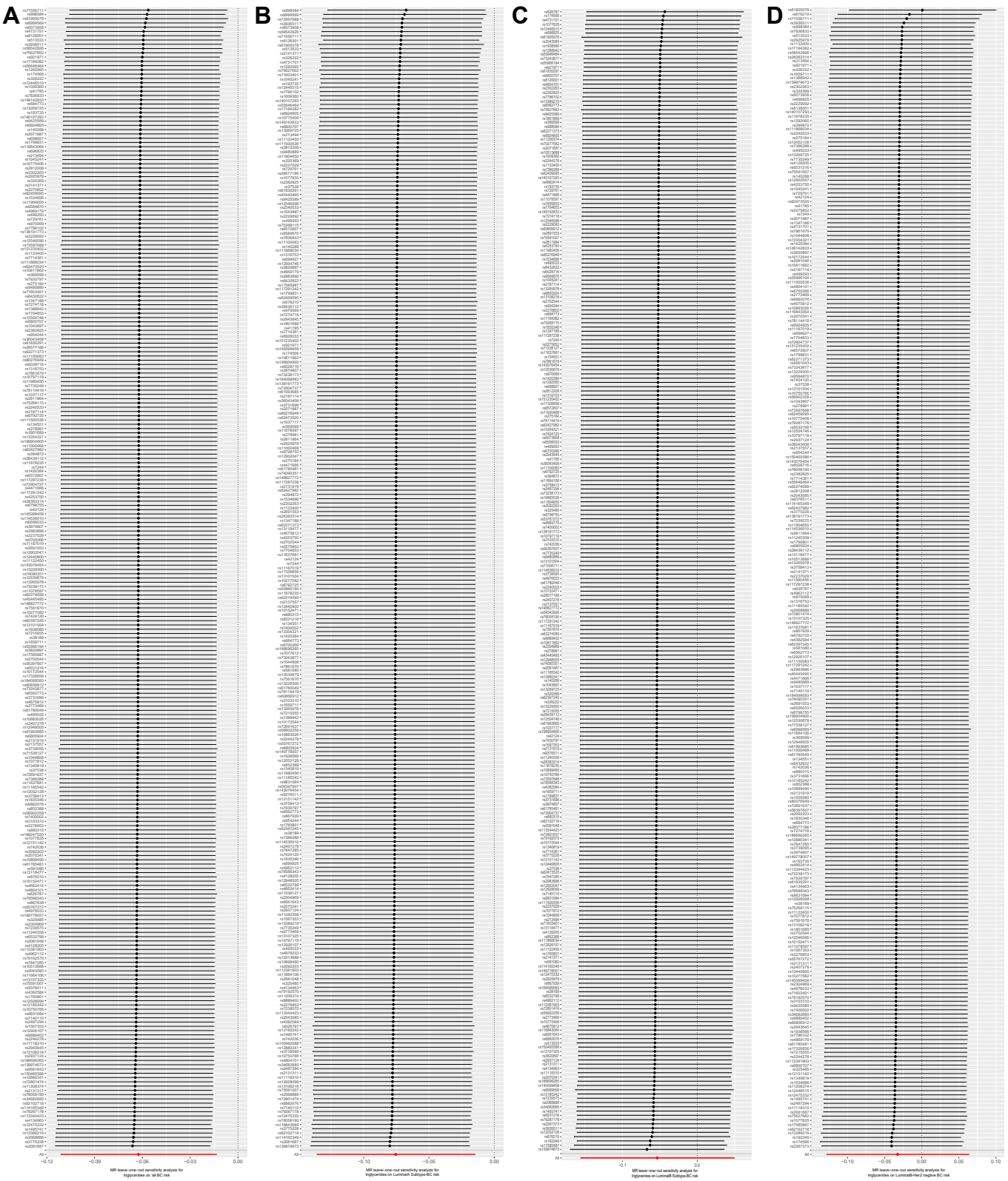

**F**

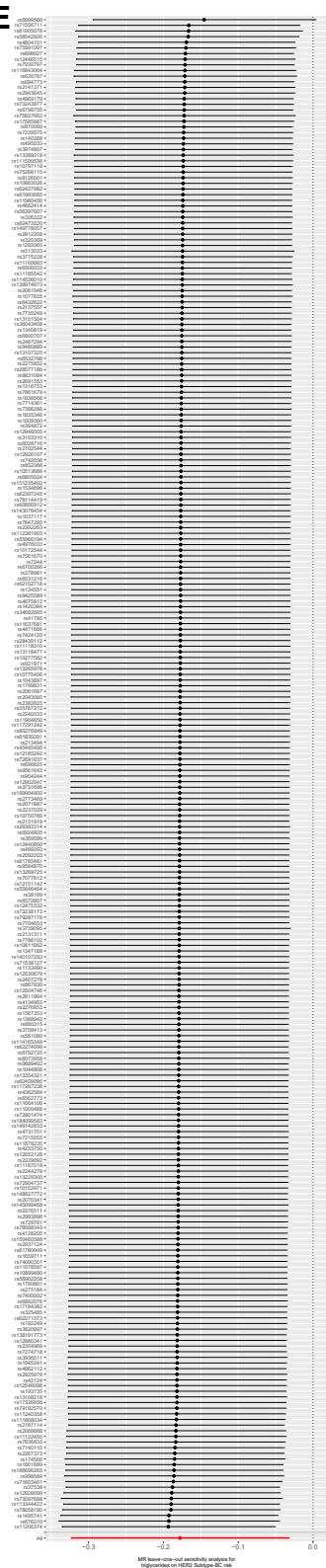**F**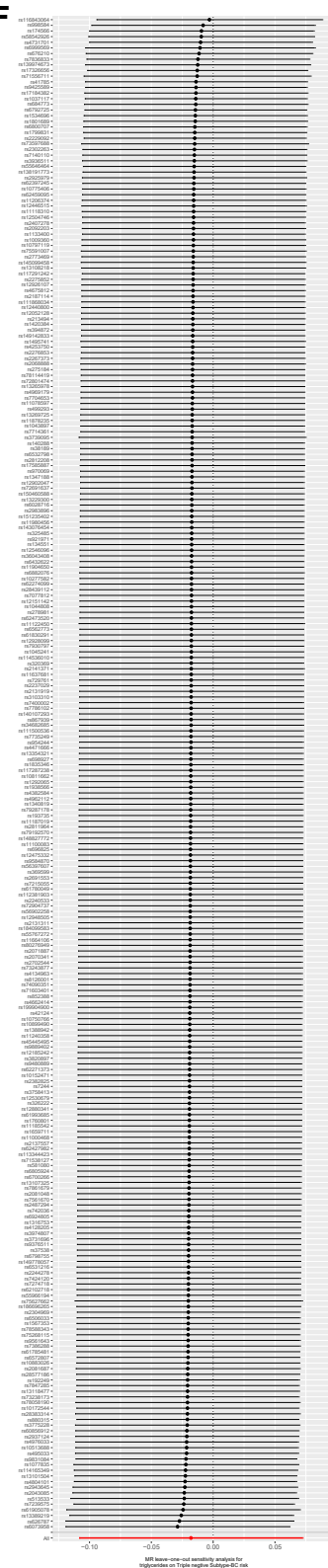

Supplement: Supplementary file 8 — Data S8. [file CAM4-14-e70698-s002.pdf]

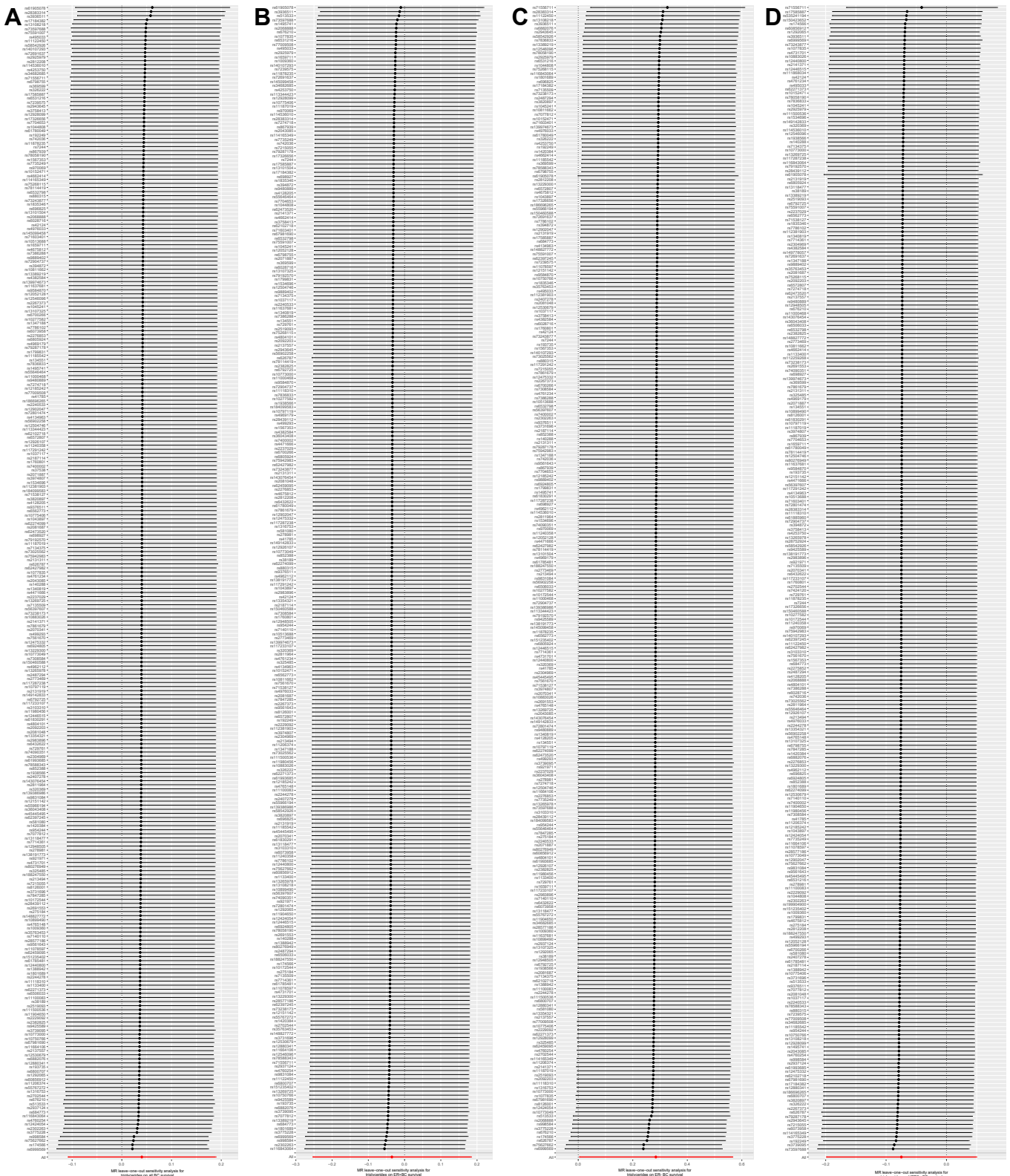

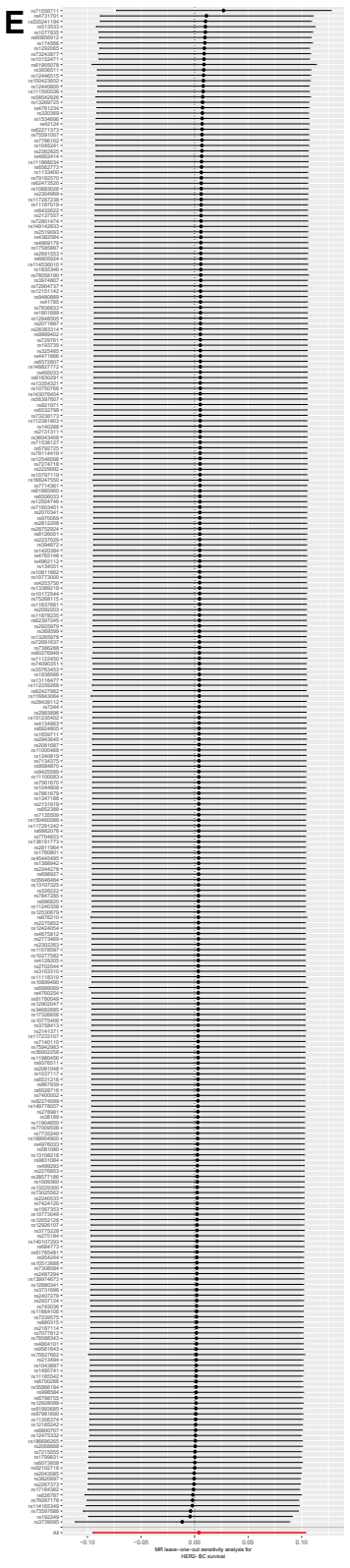

Supplement: Supplementary file 9 — Data S9. [file CAM4-14-e70698-s010.pdf]
