# Supplementary material for: Complex Role of Circulating Triglycerides in Breast Cancer Onset and Survival: Insights From Two‐Sample Mendelian Randomization Study
Source: Cancer Med. 2025 Feb 17;14(4):e70698. doi: 10.1002/cam4.70698 (PMC11831496; doi:10.1002/cam4.70698)
Supplement: Supplementary file 10 — Data S10. [file CAM4-14-e70698-s007.docx]

Additional file 10: Causal relationship of triglycerides with breast cancer and survival risk after identifying and removing SNPs with horizontal pleiotropy using MR-PRESSO testing

| Exposure | Outcome | MR Analysis | Estimate | SD | T-stat | P-value |
| --- | --- | --- | --- | --- | --- | --- |
| Triglycerides | allBC risk | Raw | -0.06251645 | 0.02488031 | -2.512688 | 0.012573569 |
|  |  | Outlier- corrected | -0.0625868 | 0.02273961 | -2.752325 | 0.006338987 |
|  | LuminalA risk | Raw | -0.07539578 | 0.03081088 | -2.44705 | 0.01505293 |
|  |  | Outlier-corrected | -0.07382129 | 0.02920743 | -2.527484 | 0.01207929 |
|  | LuminalB risk | Raw | -0.05355639 | 0.05218796 | -1.026221 | 0.3057162 |
|  |  | Outlier-corrected | -0.06896748 | 0.04911705 | -1.404145 | 0.1614464 |
|  | Her2enrich risk | Raw | -0.1779116 | 0.07418867 | -2.398097 | 0.01716733 |
|  |  | Outlier-corrected | NA | NA | NA | NA |
|  | luminalB Her2Negative risk | Raw | -0.03261431 | 0.04887878 | -0.6672487 | 0.5051895 |
|  |  | Outlier-corrected | -0.04319844 | 0.04501327 | -0.9596823 | 0.3380932 |
|  | Triple Negative risk | Raw | -0.0179662 | 0.04608922 | -0.3898135 | 0.6969853 |
|  |  | Outlier-corrected | -0.01284135 | 0.04541538 | -0.2827534 | 0.7775858 |
|  | allBC Survival | Raw | 0.04021514 | 0.07579917 | 0.5305485 | 0.5961537 |
|  |  | Outlier-corrected | NA | NA | NA | NA |
|  | ER Negative Survival | Raw | 0.2846012 | 0.143212 | 1.987272 | 0.04787341 |
|  |  | Outlier-corrected | NA | NA | NA | NA |
|  | ER Positive Survival | Raw | -0.03501487 | 0.1079109 | -0.3244795 | 0.74582 |
|  | Her2 Negative Survival | Outlier-corrected | NA | NA | NA | NA |
|  |  | Raw | 0.004115063 | 0.05065937 | 0.08123006 | 0.935317 |
|  |  | Outlier-corrected | NA | NA | NA | NA |
|  | Her2 Positive Survival | Raw | -0.07473651 | 0.06337185 | -1.179333 | 0.2392667 |
|  |  | Outlier-corrected | NA | NA | NA | NA |
