# Supplementary material for: Complex Role of Circulating Triglycerides in Breast Cancer Onset and Survival: Insights From Two‐Sample Mendelian Randomization Study
Source: Cancer Med. 2025 Feb 17;14(4):e70698. doi: 10.1002/cam4.70698 (PMC11831496; doi:10.1002/cam4.70698)
Supplement: Supplementary file 11 — Data S11. [file CAM4-14-e70698-s009.docx]

**Additional Files**

File name: Additional file 1

File format: .docx

Title: Sample sizes of overall breast cancer and its subtypes in GWAS data and according to tumor markers

Description of data: This file provides a detailed breakdown of the sample sizes for breast cancer risk data corresponding to each individual tumor marker, as well as descriptions of breast cancer subtypes based on combinations of these tumor markers.

File name: Additional file 2

File format: .pdf

Title: Genetic instruments for circulating triglycerides

Description of data:This file provides a detailed table of 299 SNPs strongly correlated with circulating triglyceride levels, chosen for the study. For each SNP, the table includes the effect allele, other alleles, effect allele frequency (EAF), beta coefficient, standard error (SE), and p-value. These SNPs were selected after rigorous screening using Phenoscanner for significant correlations with potential confounding factors. The p-values for all listed SNPs are less than 5x10^-8.

File name: Additional file 3

File format: .docx

Title: Heterogeneity testing table for causal relationship between triglyceride levels and risk of different types of breast cancer and survival prognosis

Description of data: This file presents a table for conducting a heterogeneity test using Cochran's Q test. The test explores the causal relationship between circulating triglycerides and the risk as well as survival prognosis of different breast cancer subtypes. The table highlights the Q statistic, degrees of freedom (Q_df), and associated p-values (Q_pval) for exposures and outcomes under various Mendelian randomization methods. The results contribute to assessing the potential presence of consistency and pleiotropy in the genetic instruments utilized within the Mendelian randomization analysis.

File name: Additional file 4

File format: .pdf

Title: Scatter plot of associations between triglycerides and disease risk of all breast cancer and its subtypes

Description of data: This file highlights a series of six scatter plots (Figures A-F) that depict the associations between triglyceride levels and the disease risk of various breast cancer subtypes: A: Association between overall breast cancer (all BC) risk and triglyceride levels. B: Association of Luminal A subtype breast cancer risk with triglyceride levels. C: Association of Luminal B subtype breast cancer risk with triglyceride levels. D: Association between Luminal B HER2-negative subtype breast cancer risk and triglyceride levels. E: Association of HER2-enriched subtype breast cancer risk with triglyceride levels. F: Association between Triple-negative subtype breast cancer risk and triglyceride levels. For each subtype, the associations are analyzed using four distinct Mendelian Randomization (MR) test methods: Inverse variance weighted (fixed effects)，MR Egger, Inverse variance weighted (multiplicative random effects),Weighted median.

File name: Additional file 5

File format: .pdf

Title: Scatter plot of associations between triglycerides and survival risk for all breast cancer and its subtypes

Description of data: This file contains a series of scatter plots (Figures A-E) illustrating the associations between circulating triglycerides and the survival risk for various breast cancer subtypes: A: Represents the association between overall breast cancer (all BC) survival risk and triglyceride levels. B: Represents the association between ER+ breast cancer survival risk and triglyceride levels. C: Represents the association between ER- breast cancer survival risk and triglyceride levels. D: Represents the association between HER2+ breast cancer survival risk and triglyceride levels. E: Represents the association between HER2- breast cancer survival risk and triglyceride levels. For each of these survival risks, the associations are evaluated using four distinct Mendelian Randomization (MR) test methods: Inverse variance weighted (fixed effects),MR Egger, Inverse variance weighted (multiplicative random effects),Weighted median.

File name: Additional file 6

File format: .pdf

Title: Funnel plot of relationship between causal effect of triglycerides on all breast cancer risk and its subtypes and reciprocal of the standard error of causal estimation using each individual SNP separately

Description of data: This file features funnel plots (Figures A-F) that provide a detailed representation of the causal effects of circulating triglycerides on the risk of various breast cancer subtypes. Each point on the plot represents a specific SNP. The horizontal axis (βIV) indicates the causal effect estimation for each SNP, while the vertical axis (1/SE_IV_) represents the precision of these estimates, specifically the reciprocal of the standard error of the causal effect estimation. A: Illustrates the relationship between the overall breast cancer (all BC) risk and triglyceride levels. B: Illustrates the relationship between the Luminal A subtype breast cancer risk and triglyceride levels. C: Illustrates the relationship between the Luminal B subtype breast cancer risk and triglyceride levels. D: Illustrates the relationship between the Luminal B HER2-negative subtype breast cancer risk and triglyceride levels. E: Illustrates the relationship between the HER2-enriched subtype breast cancer risk and triglyceride levels. F: Illustrates the relationship between the Triple-negative subtype breast cancer risk and triglyceride levels.

File name: Additional file 7

File format: .pdf

Title: Funnel plot of relationship between causal effect of triglycerides on the survival risk for all breast cancer and its subtypes and reciprocal of the standard error of causal estimation using each individual SNP separately

Description of data: This file features funnel plots (Figures A-E) that provide a detailed representation of the causal effects of circulating triglycerides on the survival risk for various breast cancer subtypes. These plots are used to assess the consistency and potential biases in the genetic associations between triglyceride levels and the survival risks of different breast cancer subtypes. Each point on the plot represents a specific SNP. The horizontal axis (βIV) indicates the causal effect estimation for each SNP, while the vertical axis (1/SE_IV_) represents the precision of these estimates, specifically the reciprocal of the standard error of the causal effect estimation. A: Funnel plot illustrating the relationship between overall breast cancer (all BC) survival risk and triglyceride levels. B: Funnel plot illustrating the relationship between ER+ breast cancer survival risk and triglyceride levels. C: Funnel plot illustrating the relationship between ER- breast cancer survival risk and triglyceride levels. D: Funnel plot illustrating the relationship between HER2+ breast cancer survival risk and triglyceride levels. E: Funnel plot illustrating the relationship between HER2- breast cancer survival risk and triglyceride levels.

File name: Additional file 8

File format: .pdf

Title: Leave-one-SNP-out plot for triglycerides and disease risk for all breast cancer and its subtypes.

Description of data: This file contains a series of Leave-one-SNP-out plots (Figures A-F) that visually represent the robustness of the causal relationship between circulating triglycerides and the risk of various breast cancer subtypes, based on Mendelian Randomization analysis. In these plots, the causal effect is recalculated multiple times, each time omitting one SNP, to ensure that no single SNP unduly influences the overall result. A: Leave-one-SNP-out plot illustrating the relationship between overall breast cancer (all BC) risk and triglyceride levels. B: Leave-one-SNP-out plot illustrating the relationship between Luminal A subtype breast cancer risk and triglyceride levels. C: Leave-one-SNP-out plot illustrating the relationship between Luminal B subtype breast cancer risk and triglyceride levels. D: Leave-one-SNP-out plot illustrating the relationship between Luminal B HER2-negative subtype breast cancer risk and triglyceride levels. E: Leave-one-SNP-out plot illustrating the relationship between HER2-enriched subtype breast cancer risk and triglyceride levels. F: Leave-one-SNP-out plot illustrating the relationship between Triple-negative subtype breast cancer risk and triglyceride levels.

File name: Additional file 9

File format: .pdf

Title: Leave-one SNP-out plot for triglycerides and survival risk for all breast cancer and its subtypes

Description of data: This file presents a series of "Leave-one SNP-out" plots (Figures A-E) that visually assess the robustness of the causal relationship between circulating triglycerides and the survival risk associated with various breast cancer subtypes. These plots re-evaluate the causal effect multiple times, each time excluding one SNP, to ensure that the overall result is not disproportionately influenced by any single SNP. A: Leave-one SNP-out plot illustrating the relationship between overall breast cancer (all BC) survival risk and triglyceride levels. B: Leave-one SNP-out plot depicting the relationship between ER+ breast cancer survival risk and triglyceride levels. C: Leave-one SNP-out plot showing the relationship between ER- breast cancer survival risk and triglyceride levels. D: Leave-one SNP-out plot representing the relationship between HER2+ breast cancer survival risk and triglyceride levels. E: Leave-one SNP-out plot detailing the relationship between HER2- breast cancer survival risk and triglyceride levels.

File name: Additional file 10

File format: .xlsx

Title: Causal relationship of triglycerides with breast cancer and survival risk after identifying and removing SNPs with horizontal pleiotropy using MR-PRESSO testing

Description of data: This table highlights the results of the causal relationship between circulating triglycerides and the risk and survival outcomes for various breast cancer subtypes. The findings have been refined using the MR-PRESSO testing method to identify and exclude SNPs exhibiting horizontal pleiotropy, ensuring the robustness of the results by accounting for potential pleiotropic influences. The table details the estimate of the causal effect, the standard deviation (SD) of this estimate, the T-statistic from the MR analysis, and the associated P-value. The presence of 'NA' in the outlier-corrected rows indicates that the MR-PRESSO test did not identify any significant outliers for those specific analyses.
